# Does a Barcoding Gap Exist in Prokaryotes? Evidences from Species Delimitation in Cyanobacteria

**DOI:** 10.3390/life5010050

**Published:** 2014-12-31

**Authors:** Ester M. Eckert, Diego Fontaneto, Manuela Coci, Cristiana Callieri

**Affiliations:** Microbial Ecology Group, Institute of Ecosystem Study, National Research Council, Largo Tonolli 50, 28922 Verbania, Italy; E-Mails: d.fontaneto@ise.cnr.it (D.F.); m.coci@ise.cnr.it (M.C.); c.callieri@ise.cnr.it (C.C.)

**Keywords:** cyanobacteria, DNA barcoding, taxonomy, operational taxonomic units, species in prokaryotes

## Abstract

The amount of information that is available on 16S rRNA sequences for prokaryotes thanks to high-throughput sequencing could allow a better understanding of diversity. Nevertheless, the application of predetermined threshold in genetic distances to identify units of diversity (Operative Taxonomic Units, OTUs) may provide biased results. Here we tests for the existence of a barcoding gap in several groups of Cyanobacteria, defining units of diversity according to clear differences between within-species and among-species genetic distances in 16S rRNA. The application of a tool developed for animal DNA taxonomy, the Automatic Barcode Gap Detector (ABGD), revealed that a barcoding gap could actually be found in almost half of the datasets that we tested. The identification of units of diversity through this method provided results that were not compatible with those obtained with the identification of OTUs with threshold of similarity in genetic distances of 97% or 99%. The main message of our results is a call for caution in the estimate of diversity from 16S sequences only, given that different subjective choices in the method to delimit units could provide different results.

## 1. Introduction

The question of whether species are a pervasive phenomenon of life, or whether they exist only in sexual eukaryotes is still open and may even never find an answer. The reliability of species as independent evolutionary units subsumes the existence of a cohesive force that maintains organisms relatively homogeneous within species in their genetics, ecology, morphology, physiology, *etc.* and also maintains the organisms separated between species [1,2,3,4]. Such cohesive force in sexual organisms is hypothesized to be sexual recombination: gene flow within species keeps the species pool homogeneous, whereas reproductive isolation between species maintains species as independently evolving units [5].

In prokaryotes, horizontal gene transfer, the exchange of genes between different species, is quite common [6,7]; this should have the consequence that species do not exist, but a gradient of differences should be found with unclear genetic boundaries between taxonomic units [8]. Nevertheless, prokaryote taxonomy works, different species can be identified and have species-specific features that allow researchers to identify them using their genetics, physiology, ecology and even morphology, with a clear framework of species concept [9,10,11,12,13]. Thus, cohesive forces other than sexual recombination may be present to originate and maintain species, even if prokaryotic sex-like recombination exists [14]. One of the main hypotheses is that ecological sweeps may act in prokaryotes and are able to produce the same patterns of independent evolutionary units [11,15,16,17]. Based on these assumptions, two concepts for the existence of species have developed for prokaryotes, based either on ecological divergence or on barriers to recombination; both processes could generate independently evolving groups that are equivalent to species in sexual eukaryotes [18].

Regardless of the mechanism that produces independent units, of the interaction of different mechanisms, and of the philosophical concept that is the mind of the researcher when defining species, taxonomy using DNA sequences in prokaryotes is traditionally and empirically mostly based on similarity in 16S sequences [19]. The main assumption of the methods is that individuals of the same species will have similar sequences, whereas individuals of different species will have a lower proportion of similarity. The most common quantitative approach in DNA taxonomy is based on a predetermined fixed threshold in genetic distances of sequences of 16S rRNA (16S), ranging from 97% to 99%, to identify operational taxonomic units called OTUs [20,21,22,23].

**Figure 1:**
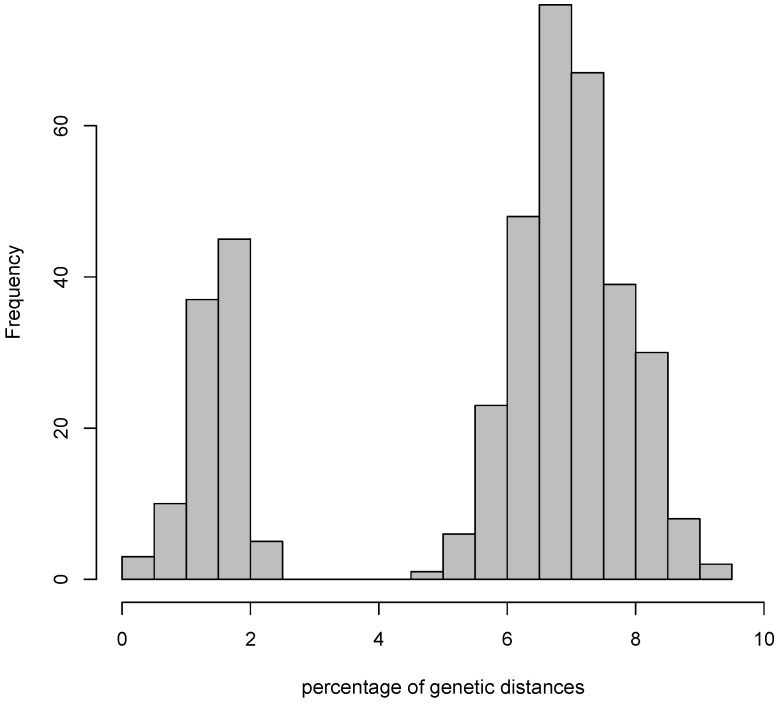
Plot of the distribution of pairwise genetic distances in a hypothetical group of organisms of different species. All the genetic distances that belong to pairwise comparisons of organisms within the same species fall in the bars on the left, in this case below 2.5%; all the distances that belong to pairwise comparisons between organisms of different species fall in the bars on the right, in this case between 4.5% and 9.5%; no intermediate distances exist between the two distributions, defining a dataset-specific barcoding gap ranging from 2.5% to 4.5%. In this case, a 97% threshold would provide reliable units of diversity, whereas a 99% threshold would overestimate the actual biological diversity.

Enormous progress happened in prokaryote taxonomy in the recent years [8,12,24]. However, the data produced with novel methodologies, such as next generation sequencing, often requires a high throughput taxonomic classification of sequences, such as fixed threshold to identify OTUs. This kind of fixed numeric classification can always only be a vague approximation to the actual structure of relatedness of organisms. Surprisingly, only few tests have been performed to assess whether a barcoding gap, similar to the one commonly found in DNA barcoding markers in animals [25], actually exists in prokaryotes (e.g., [26,27]). The existence of a barcoding gap would mean that genetic distances within each species are short, genetic distances between species are long, and no intermediate distances are present (Figure 1). A test on the existence of such barcoding gap in prokaryotes will help researchers deciding which threshold to use and whether separate units can be actually found using 16S. The untested application of fixed thresholds (97% or 99%) in genetic distances will always produce separate units, regardless of whether they are meaningful or not; on the other hand the existence of a barcoding gap will show that such units are biological reality.

The main aim of this study is thus to empirically test whether a barcoding gap can be easily identified in 16S in prokaryotes or not. We choose Cyanobacteria as an example of prokaryotes, not because they are representative for all prokaryotes, but because there is ample phenotypic, ecological, physiological ultrastructural, and biochemical evidence of the existence of independently evolving units in this group [28,29]. Thus, the expectation is that, if a barcoding gap exists in prokaryotes, this should be more easily seen in taxa where groups can be identified also with other methods, as in Cyanobacteria. Our first step is thus to test for the existence of a barcoding gap across a large selection of Cyanobacteria genera, in order to provide evidence for the existence of genetic patterns of independent evolution of 16S between species. After testing for the barcoding gap, we compare the results of our tests with the outcome of traditional OTU delimitation on the same dataset and discuss specific cases highlighting congruencies and incongruencies. We acknowledge in advance that taxonomy performed relying on a single locus has strong limitations, due for example to unlinked variation among loci and to potential horizontal gene transfer. Nevertheless, differences in 16S sequences remain the common tool used in large-scale diversity studies (e.g., [30,31]), and thus our results may have a profound influence on the way researchers approach the study of large amount of 16S data, especially with the widespread application of metagenetic surveys from high-throughput sequencing. Our aim was not to solve any taxonomic problem in specific genera, but to gather reliable genetic information from monophyletic clusters, loosely corresponding to named entities, such as species complexes, genera or groups of closely related genera, in order to search for the existence of a barcoding gap.

## 2. Experimental Section

### 2.1. Selection of Sequences

We aimed at selecting carefully edited, checked and pre-aligned near full-length 16S sequences for which as little errors as possible would be present. Thus, we chose to retrieve aligned sequences of Cyanobacteria from the SILVA database for ARB release 111 [32]. Monophyletic clades of sequences richer than 30 sequences were selected in the provided tree using the software package ARB [33] and retained if all the sequences belonged to a taxonomic rank encompassing one or two closely related genera or a species complex. Thereby a group was considered monophyletic if it was monophyletic in the tree provided with the database, regardless of the taxonomy and the nomenclature of the organisms included in the clade (Figure S1), and only secondarily if there was a correspondence with known taxa.

The names of genera and species used in this study where copied form the names given in the database. This choice was made for the reason that cyanobacterial phylogeny underlies continuous changes and genera and species are often reclassified (e.g., [34]). Thus a whole different type of work would be required in order to use all state-of-art classification of cyanobacteria. Moreover, for the aims of this study not so much the phylogenetic classification of the bacterial species, but the genetic structure of the cyanobacterial 16s rRNA genes was of importance. The use of the names provided in the database on the other hand enables other researchers to conduct a similar analysis using the same sequences. Thus some old and revised names are used, for example the species name *Anabaena bergii* and *Aphanizomenon ovalisporum* are used throughout the study despite its revised taxonomy in the genus *Chrysosporum* [35].

Fifteen monophyletic clades were identified (Figure S1); moreover, we also analysed one subclade within the largest of the clades, including only the monophyletic *Prochlorococcus* within the clade of *Prochlorococcus*-*Synechococcus*. Sequences termed *Synechococcus* on the other hand were analyzed together with sequences of the monophyletic clade *Prochlorococcus*, since *Synechococcus* is known to be a polyphyletic group and if all sequences (including a minimum of five lineages [36,37,38]) are taken together, *Prochlorococcus* has to be included, too.

As an additional control and in order to avoid biases introduced by chimeras 234 sequences with Silva pintail values lower than 70 were excluded [39]. Aligned sequences for each of the 16 clades (accession numbers Table S1) were exported into separate fasta files that were then used for the following analyses on testing for the presence of a barcoding gap and on defining traditional OTUs through *a-priori* thresholds. By using aligned sequences from SILVA, we avoided potential biases introduced by differences in the algorithms that could be applied for the alignment of ribosomal markers [40].

### 2.2. Testing for the Barcoding Gap

We applied a recently developed method in DNA taxonomy in animals, the Automatic Barcode Gap Detector (ABGD: [41]), able to test for the existence of a barcoding gap in a dataset of genetic sequences of a single marker obtained from different individuals from closely related species. Instead of using an *a-priori* predefined threshold in genetic distances to delimit species as it is done when applying a 97% or a 99% threshold, the method searches for an optimal threshold in the dataset, and then uses such dataset-specific threshold to delimit species. The method starts from a dataset of aligned sequences, produces a distance matrix, and ranks all the distances. Then, it looks for the first significant increase in pairwise distance indicative of a transition from intraspecific to interspecific relationships, with the possibility to define a minimum user-defined range of prior intraspecific divergence, in order to exclude potentially misleading inter-population differences. Species are then delimited using the most likely threshold obtained by the analysis. The ABGD has been used predominantly to delimit species in animals until now, and in most cases it has been found to delimit groups identical or similar to more theory-based and potentially more accurate methods in animal DNA taxonomy [42,43,44]. Thus, its application in prokaryotes to look for the presence of a barcoding gap would be very useful.

In our case, first we tested if a barcoding gap could be actually found in each of the 16 datasets, and, in the cases with a positive assessment, we estimated how many species could be identified in the dataset. In order to avoid ambiguities in terminology, we refer to species delimited with this method as to “ABGD units”. Such units could be formed by clusters of similar sequences or by single sequences. These singletons could be due to erroneous sequences, given that they are unique sequences [45], thus, we reported both the total number of units and the number of clusters, excluding singletons. The comparison between the two numbers could also be used as a measure of under-sampling for each dataset: a higher number of singletons would imply a higher level of under-sampling.

For a selection of the datasets in which a barcoding gap was found, we provided additional phylogenetic analyses, in order to test whether monophyletic clades from distance matrices resulted monophyletic also in carefully reconstructed phylogenies, and to graphically show the results. We used the built in RAxML of ARB [46] to obtain phylogenetic reconstructions through Maximum Likelihood using the same sequence alignments for the selected groups and three sequences of *Phormidium* as an outgroup. We used the default settings of RAxML, with the GTRMIX rate distribution model and the “rapid bootstrap” algorithm. Bootstrap replicates were run 1000 times.

### 2.3. Traditional OTUs Delimitation

We tested whether the ABGD method would provide results and units of diversity compatible with what can be found with the commonly applied routines in DNA taxonomy of prokaryotic 16S sequences. In order to do so, sequences from each group were clustered into OTUs using the next generation sequencing analysis pipeline Mothur version 1.33.3 for Windows [47]. Using all default settings of Mothur, sequences were filtered, a distance matrix was calculated and OTUs were generated through average neighbor algorithm. Here we report the results for OTUs clustered on a 99% and 97% identity level, since these two values are most commonly used in microbial ecology [10,20,21,22]. We will call these clusters as OTU 97% and OTU 99% units throughout the manuscript. Similarly to the ABGD units, also OTUs can be identified in clusters or singletons [45], and we reported both the total number of units and the number of clusters excluding singletons.

### 2.4. Assessing for Explanations and Confounding Factors

First of all, we wanted to test the effect of the number of sequences on the possibility to identify a barcoding gap. The rationale is that if several sequences are included in the dataset, those will either (1) fill the misleading gaps, which are present only due to under-sampling; or (2) provide such a large amount of diversity that the heterogeneous evolutionary rates in different clades in the dataset will mask the actual barcoding gap. If the number of sequences in each dataset would have a significantly negative effect, the two scenarios describing its potential effect can be disentangled by the assessment of whether the maximum genetic difference in the dataset will also have an effect, regardless of the number of sequences: if only datasets with large maximum genetic distances do not produce a barcoding gap, the latter scenario would be more plausible, whereas if no effect of maximum genetic distances is found, the first scenario would be more plausible. To address this issue, we used a logistic regression with success or failure to detect a barcoding gap in each dataset through ABGD as a response variable, and number of sequences, maximum genetic distances in the same dataset, and the interaction of number of sequences and maximum genetic distances as explanatory variables. An additional variable included in the models was the shape of the cells: the Cyanobacteria tested were either coccoid or filamentous and the different shapes might influence the interaction between individuals, potentially affecting the probability of ecological sweeps or genetic exchange [48]. We applied a generalized linear model (GLM) with binomial distribution [49] in R 2.15.0 [50].

Then, we also wanted to test whether the number of identified taxa, either as ABGD units or as OTUs from *a-priori* thresholds of 97% and 99%, simply depended on the number of sequences in the dataset. To do so, we performed a GLM with the number of taxa as response variable and the number of sequences as the explanatory variable, using the appropriate Poisson error structure for count data [49].

Additionally, we compared the results of estimates of taxonomic units from ABGD, 97% and 99% thresholds with simple correlation tests in R.

## 3. Results

### 3.1. Testing for the Barcoding Gap

**Figure 2:**
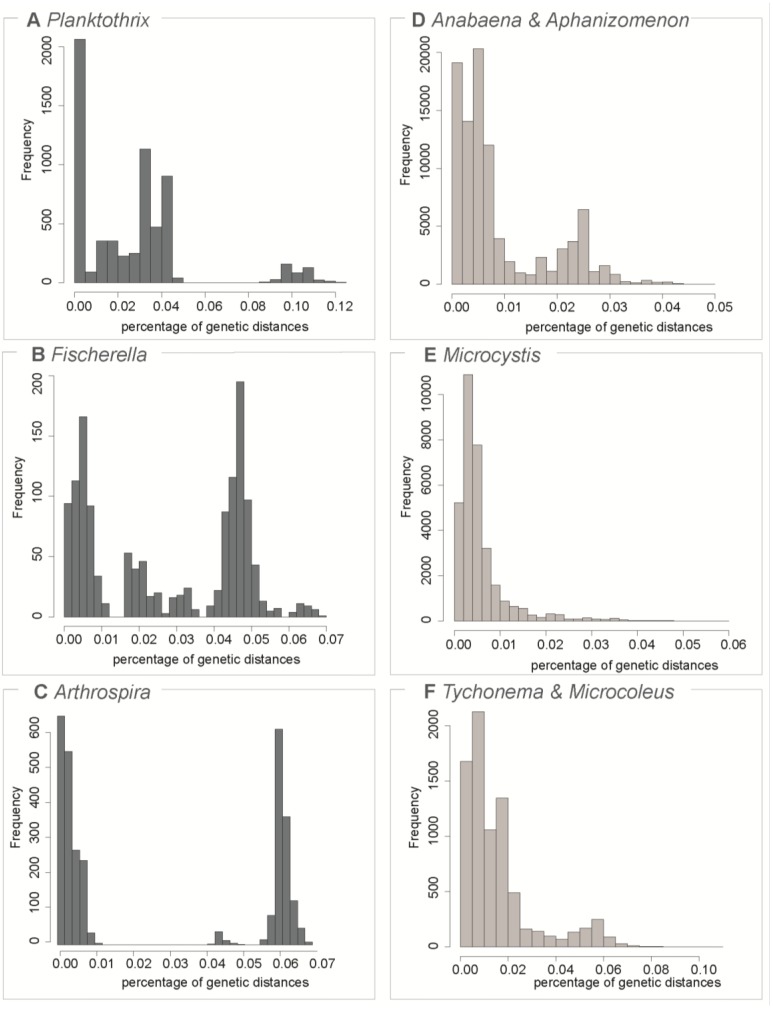
Plot of the distribution of pairwise genetic distances in six of the 16 datasets. The three datasets on the left (**A**–**C**) provided evidence of a barcoding gap through Automatic Barcode Gap Detector (ABGD), whereas no barcoding gap could be found the three datasets on the right (**D**–**F**). Note the different scale bars of the figures.

All 16 datasets were tested to see whether the presence of a barcoding gap could be identified. For seven of the 16 tested datasets a barcoding gap could be identified (Table 1, Figure 2, Figure S2) and the sequences could thus be grouped into species. The barcoding gap existed for example in *Planktothrix*, *Fischerella* and *Arthrospira* (Figure 2), whereas it was not found for *Anabaena-Aphanizomenon*, *Microcystis* and *Tychonema-Microcoleus*.

### 3.2. The Datasets

The analyzed datasets encompassed a large diversity within Cyanobacteria and included from 34 to over 2400 sequences, for a total of 4887 sequences; genetic diversity within each dataset ranged from 3% to 26% (Table 1). For more than 60% of the sequences the isolation source could be retrieved: the majority of the sequences were isolated from seawater samples (35%), followed by freshwaters (12%), biofilm samples including cyanobacterial mats (7%), epilithon soil samples (4.5%), symbiont of sponges and other invertebrates (2%).

**Table 1:** Summary of the total number of sequences and maximum genetic diversity for each of the 16 datasets, with the existence or not of a clear barcoding gap found through Automatic Barcode Gap Detector (ABGD), the number of units identified by the dataset-specific threshold of ABGD, and the number of Operative Taxonomic Units (OTUs) identified by the 97% and 99% *a-priori* threshold in genetic diversity. Numbers between brackets identify the number of units when excluding singletons.

Dataset	Number of Sequences	Maximum Genetic Diversity	Barcoding Gap	ABGD Units	OTU 97%	OTU 99%
*A. ovalisporum & A. bergii*	34	0.03	yes	2 (2)	2 (2)	6 (6)
*Anabaena & Aphanizomenon*	508	0.05	no	-	5 (4)	33 (28)
*Arthrospira*	97	0.07	yes	3 (2)	3 (3)	7 (7)
*Calothrix*	71	0.13	no	-	8 (5)	34 (13)
*Chroococcidiopsis*	88	0.11	no	-	4 (3)	25 (9)
*Cylindrospermopsis*	157	0.14	yes	2 (2)	3 (3)	25 (18)
*Fischerella*	58	0.07	yes	6 (4)	2 (2)	4 (4)
*Leptolyngbya & Chamaesiphon*	400	0.26	no	-	8 (3)	153 (67)
*Microcystis*	316	0.06	no	-	2 (2)	11 (7)
*Nodularia*	74	0.05	yes	2 (2)	4 (2)	12 (6)
*Nostoc*	323	0.04	no	-	5 (4)	19 (14)
*Phormidium*	70	0.16	yes	5 (4)	3 (2)	24 (8)
*Planktothrix*	108	0.12	yes	13 (6)	3 (3)	10 (9)
*Prochlorococcus*	1011	0.05	no	-	3 (2)	29 (13)
*Prochlorococcus & Synechococcus*	2448	0.17	no	-	40 (18)	286 (99)
*Tychonema & Microcoleus*	135	0.108	no	-	1 (1)	12 (7)

Some of the datasets were represented by a single monophyletic genus, e.g., *Arthrospira*, *Planktothrix*, and *Nostoc*, others were represented by two genera that were monophyletic only when taken together in the database used, e.g., *Leptolyngbya* and *Chamaesiphon*, whereas others were represented by species complexes, e.g., *Aphanizomenon ovalisporum* and *Anabaena bergii*. A special case is that of *Prochlorococcus*-*Synechococcus*: this dataset was included in two different forms, first as the whole monophyletic clade with its 2448 sequences, and then also as a nested monophyletic subclade represented only by the 1011 sequences of *Prochlorococcus*. In order not to bias the statistical analyses by including the same sequences twice in two datasets, we performed all the analyses first with all 16 datasets, and then controlled for the bias by disregarding the *Prochlorococcus*-*Synechococcus* dataset and using only the remaining 15 datasets.

### 3.3. Traditional OTUs Delimitation

All datasets encompassed a diversity that could be subdivided using 97% and 99% thresholds, and the number of OTUs ranged from 2 to 18 and from 4 to 99, respectively, when excluding singletons (Table 1). The amount of OTUs was significantly affected by the number of sequences included in the datasets for both thresholds, both when including or excluding singletons (Table 2); the significant effect was present also when excluding the *Prochlorococcus*-*Synechococcus* dataset (results not shown).

**Table 2:** Results of the statistical assessments of various explanatory variables for the different hypotheses explicitly tested in the study. (**A**) Generalized Linear Model (GLM) with binomial error for the existence of a barcoding gap as a function of the number of sequences and the maximum genetic diversity in each of the 16 datasets; (**B**); (**C**); and (**D**) GLM with Poisson error for the number of Automatic Barcode Gap Detector (ABGD) units, Operative Taxonomic Units (OTUs) from 97% and OTUs from 99% threshold as a function of the number of sequences. The results from estimates of diversity obtained when excluding singletons are reported between brackets.

**(A) Presence of a Barcoding Gap**	**Estimate**	***p***
(intercept)	−14.19 ± 566.51	0.898
Number of sequences	−0.03 ± 0.02	0.286
Maximum genetic diversity	−22.3 ± 33.3	0.504
Number of sequences: Maximum genetic diversity	0.08 ± 0.14	0.578
Shape	19.66 ± 566.1	0.897
**(B) ABGD units**	**Estimate**	***p***
(intercept)	1.35 ± 0.44 (1.13 ± 0.53)	0.002 (0.035)
Number of sequences	0.00 ± 0.00 (0.00 ± 0.00)	0.624 (0.979)
**(C) OTUs 97%**	**Estimate**	***p***
(intercept)	1.06 ± 0.15 (0.87 ± 0.17)	<0.0001 (<0.0001)
Number of sequences	0.00 ± 0.00 (0.00 ± 0.00)	<0.0001 (<0.0001)
**(D) OTUs 99%**	**Estimate**	***p***
(intercept)	2.99 ± 0.05 (0.22 ± 0.07)	<0.0001 (<0.0001)
Number of sequences	0.00 ± 0.00 (0.01 ± 0.00)	<0.0001 (<0.0001)

### 3.4. Comparison of ABGD Units and OTUs

The number of “species” detected as ABGD units was not related to the number of OTUs found with the 97% (*t* = 0.25, *p* = 0.81) nor the 99% threshold (*t* = 0.33, *p* = 0.75): thus, applying a dataset-specific threshold provides estimates of diversity that are not comparable to the traditional OTUs; moreover, ABGD found a number of units that was always lower than the number of OTUs. The two thresholds to detect OTUs, 97% and 99%, provided estimates that were correlated (*t* = 5.46, *p* < 0.001), with the number of OTUs being always higher when a higher threshold was considered (Table 1). The results were qualitatively confirmed when excluding the *Prochlorococcus*-*Synechococcus* dataset (results not shown).

**Figure 3:**
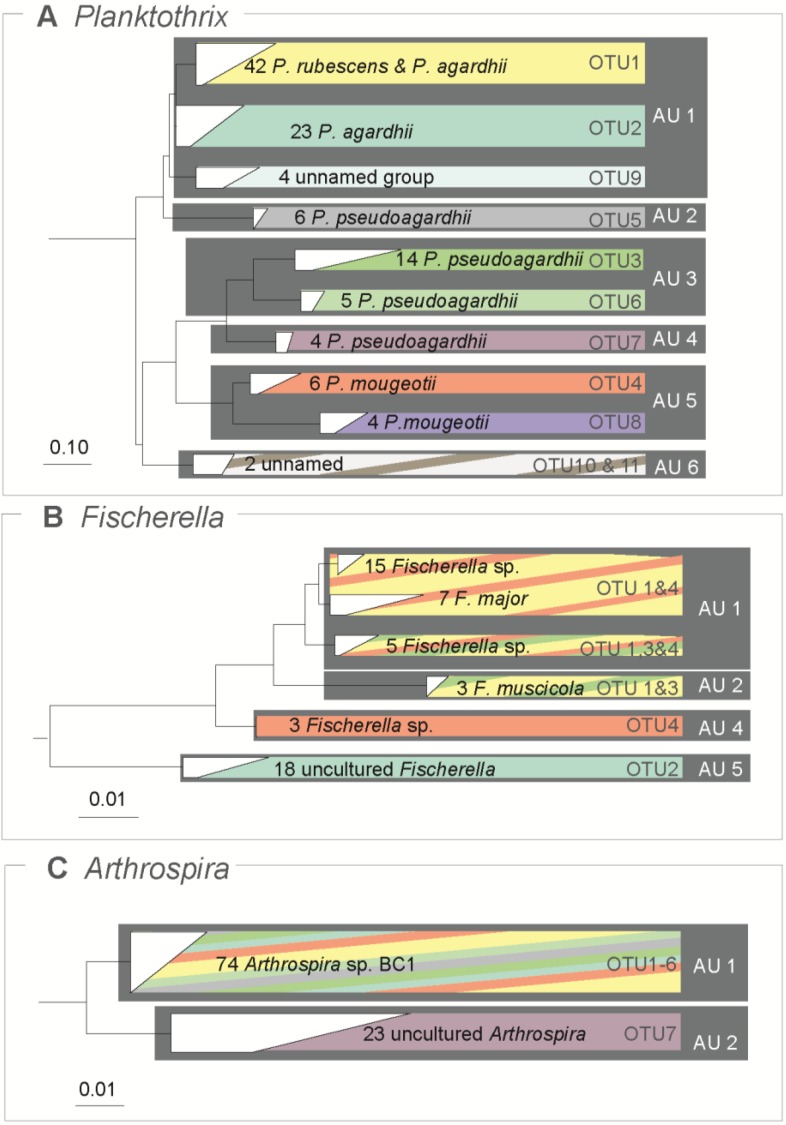
Rooted Maximum Likelihood phylogenetic trees for three datasets with barcoding gap presented in Figure 2. Scale bars represent the number of substitutionns per site according to the GTRMIX model. All branches depicted are supported by bootstrap values of 100%. The numbers represent unique sequences within the clade. Colors within each bar correspond to 99% identity Operative Taxonomic Units (OTUs) within the collapsed monophyletic clades and the dark grey squares represent ABGD units (AU). The names correspond to the ones from SILVA database 111 [32].

In order to visually compare the identification of ABGD units and OTUs, we visualized the phylogenetic reconstructions for *Planktothrix*, *Fischerella* and *Arthrospira*, the three genera where a barcoding gap was unambiguously found. In all three cases the ABGD units corresponded completely with monophyletic phylogenetic groups on the trees (Figure 3). In the case of the genus *Planktothrix*, the 99% OTUs corresponded with monophyletic groups nested within ABGD units. For example, ABGD unit 1 (AU1 in Figure 3A) consisted of three OTUs. In contrast, all ABGD units but not all OTUs resulted monophyletic in *Arthrospira* and *Fischerella*. Three OTUs in *Fischerella* where distributed within two different monophyletic groups corresponding to two ABGD units (Figure 3B). For *Arthrospira*, six OTUs were mixed within the same monophyletic group corresponding to one ABGD unit (Figure 3C).

## 4. Discussion

Interestingly, even prokaryotes as Cyanobacteria have significant evidence of a barcoding gap in at least seven out of 15 datasets that we analyzed. Such barcoding gap is not a sampling artifact, due to the lack of effect of sample size, and can be considered a reliable representation of the biological reality of those datasets. Thus, even on the basis of 16S in prokaryotes there are units that can be identified through differences in genetic distances, as predicted by theoretical expectations from the existence of independently evolving species [11,15,18,26,51].

At present, we have no explanations on the reason for the differential response among the Cyanobacteria genera and clades tested in our exercise. A barcoding gap was found in half of the cases, with no clear pattern on the success or failure in identifying it. For example, we clearly observed a barcoding gap in differences in 16S defining six units in *Planktothrix.* Considering the naming of *Planktothrix* sequences in the database, some OTUs and ABGD units seem to correspond not only to monophyletic groups but also to named species, such as *P. mougeotii* or similarly in the case of *Fischerella muscicola* (Figure 3). On the contrary, in the *Prochlorococcus*-*Synechococcus* clades no distinction between within-species and between-species genetic distances could be found. One possibility is that clades showing a “fan like” tree topology, such as *Prochlorococcus*-*Synechococcus* indicate a rapid adaptive radiation, which may mask the evolutionary pattern of a barcoding gap [45,52]. No evidence for the effect of cell shape could be found, but this lack of significance could be due to the low power of the analysis: only three datasets (*Chroococcidiopsis*, *Microcystis* and *Prochlorococcus* + *Synechococcus*) out of 15 were coccoid and for none of them a barcoding gap was found. Only additional datasets would enable us to accurately test for the effect of cell shape. Moreover it has to be emphasized that a close analysis of the actual properties of the cyanobacterial groups tested is limited by the fact that we used the provided names for the sequences and groups. Thus this study intends only to verify the existence of a barcoding gap within the 16S rRNA sequences of certain cyanobacterial groups, and by no means revise or confirm the complex cyanobacterial taxonomy.

Regardless of the cohesive mechanisms that can create the barcoding gap [15,18,51], and of the potential causes of the failure in identifying it in specific datasets, the results from the ABGD tool and from the traditional approach in delimiting OTUs are not comparable and provide different estimates of diversity. Our assessment has thus two main messages for the use of 16S sequences to describe diversity in Cyanobacteria. The first one is that estimates of diversity from the same data may provide rather different numbers, depending on the rationale that is applied to the analyses. The Automatic Barcode Gap Detector and the 97% and 99% thresholds to define units of diversity (OTUs) provide numbers that cannot be compared. Thus, no unique simple and easy tool from DNA taxonomy will be able to provide a structure in the analysis of biological diversity in cyanobacteria. This is an unfortunate situation and the subjective choices on which method to adopt in assessing diversity and richness from large data obtained from high-throughput sequencing will produce more confusion instead of more clarity. This is of particular importance if also taken into consideration that the sequences used in this study where near full length sequences, and the accurate determination of taxonomic and phylogenetic units is even more difficult with short sequences as derived from next generation sequencing [12]. The second message is a direct consequence of the first one, and is that we advise not to blindly use any DNA barcoding method in 16S to identify species in Cyanobacteria, but to look for coincidences and correlations between DNA barcoding through e.g., the Automated Barcode Gap Discovery as applied in the study. However, an accurate taxonomy can never be achieved by the use of a barcoding method only, since it is based on nucleotide substitutions of a single gene. Accurate classification always requires an integrative taxonomy effort including characteristics from ecology, morphology and physiology, as already previously suggested for prokaryotes [9,28,29,45], and as it is becoming common also for animal taxonomy (e.g., [53,54]).

## Supplementary Materials

Supplementary materials can be accessed at: http://www.mdpi.com/2075-1729/5/1/50/s1.
