# Supplementary material for: Does a Barcoding Gap Exist in Prokaryotes? Evidences from Species Delimitation in Cyanobacteria
Source: Life (Basel). 2014 Dec 31;5(1):50–64. doi: 10.3390/life5010050 (PMC4390840; doi:10.3390/life5010050)
Supplement: Supplementary file 1 [file life-05-00050-s001.pdf]

Supplementary Materials

Table S1. List of all the accession numbers of the sequences used in the study.

Accession number	Accession number	Accession number	Accession number
AACY020162006	AB035551	AB045943	AB088375
AACY020370060	AB035549	AB045948	AB088405
AACY020331499	AB035550	AB045955	AB093488
AACY020482756	AB039019	AB045920	AB094351
AACY020481185	AB039003	AB045972	AB094352
AACY020482534	AB039002	AB045932	AB098071
AACY023388742	AB045902	AB045944	AB074509
AACY023390641	AB045914	AB045956	AB101003
AACY023835985	AB045926	AB045968	AB115467
AACY023901975	AB045938	AB035553	AB115474
AACY023863167	AB045950	AB045898	AB115486
AACY023868251	AB045904	AB045910	AB113665
AACY023864655	AB045916	AB045922	AB115471
AACY023870739	AB045901	AB045934	AB115473
AACY023938192	AB045928	AB045946	AB115468
AACY023936563	AB045913	AB045958	AB115483
AACY024098958	AB045940	AB045905	AB115485
AACY023937770	AB045925	AB045970	AB115480
AACY024109631	AB045952	AB045917	AB115966
AAOK01000001	AB045937	AB045941	AB115476
AANO01000009	AB045949	AB045965	AB115488
AAVW01000053	AB035552	AB045918	AB115475
AB003169	AB045899	AB045930	AB115487
AANP01000006	AB045911	AB045942	AB115472
AB001724	AB045923	AB045954	AB115470
AB003163	AB045935	AB045966	AB115484
AB012337	AB045903	AB012327	AB115482
AB012334	AB045947	AB012339	AB115466
AB012332	AB045915	AB058265	AB115478
AANO01000001	AB045959	AB058264	AB115469
AB012331	AB045900	AB058226	AB115481
AB012329	AB045927	AB058205	AB115479
AB012333	AB045971	AB058250	AB093487
AB012326	AB045907	AB074508	AB113666
AB012338	AB045912	AB074507	AB115477
AB012330	AB045919	AB039016	AB115489
AB003165	AB045939	AB045897	AB154318
AB015058	AB045951	AB045909	AB154315
AB012328	AB045924	AB045921	AB154316
AB012340	AB045896	AB045933	AB154314
AB012335	AB045931	AB045945	AB179527
AB012336	AB045936	AB045957	AB183570
AATZ01000004	AB045908	AB045969	AB183569

Table S1. *Cont.*

Accession number	Accession number	Accession number	Accession number
AB183572	AB551471	AB610889	AF062637
AB245143	AB551438	AB610896	AF067819
AB251863	AB551482	AB610895	AF067818
AB251864	AB551483	AB610887	AF076161
AB251861	AB551450	AB610891	AF076159
AB251860	AB551443	AB610894	AF076160
AB251862	AB551462	AB610888	AF076158
AB251859	AB551455	AB610892	AF053397
AB271211	AB551474	AB630389	AF053396
AB271212	AB551467	AB630390	AF076165
AB275350	AB551479	AB630387	AF091150
AB275353	AB551445	AB630388	AF098374
AB275348	AB551457	AB630385	AF098373
AB275352	AB551439	AB630384	AF098371
AB275349	AB551469	AB630391	AF098372
AB275351	AB551451	AB630681	AF098370
AB275347	AB551449	AB630686	AF115271
AB305067	AB551448	AB630683	AF115270
AB325536	AB551481	AB630687	AF076163
AB325714	AB551463	AB630678	AF132788
AB355041	AB551461	AB630680	AF076164
AB428654	AB551473	AB630677	AF132793
AB486181	AB551444	AB630685	AF132930
AB486546	AB551472	AB630684	AF081834
AB486559	AB551456	AB630679	AF139293
AB486736	AB559009	AB668059	AF139305
AB486773	AB558957	ABSE01000018	AF139317
AB486983	AB607198	ABYK01000016	AF139329
AB488091	AB607197	ABSE01000018	AF139292
AB488124	AB607194	ACDW01000004	AF139299
AB519661	AB607206	ACNY01000003	AF139295
AB551441	AB607203	ACYA01000016	AF139304
AB551442	AB607200	ACYA01000026	AF139311
AB551453	AB607204	ACYA01000056	AF139307
AB551440	AB607199	ACYB01000037	AF139323
AB551465	AB607202	ACYB01000022	AF139319
AB551447	AB607205	AE017126	AF139302
AB551466	AB607201	AF001480	AF139314
AB551446	AB607196	AF001479	AF139326
AB551452	AB607195	AF001477	AF139300
AB551477	AB608023	AF027653	AF139294
AB551478	AB610885	AF001478	AF139316
AB551464	AB610886	AF027654	AF139312
AB551476	AB610890	AF053398	AF139303
AB551470	AB610893	AF053399	AF139328

Table S1. *Cont.*

Accession number	Accession number	Accession number	Accession number
AF139324	AF247588	AF311217	AF448066
AF139306	AF247584	AF311291	AF448065
AF139315	AF247590	AF317078	AF448067
AF139318	AF247596	AF317072	AF448070
AF139327	AF247577	AF317075	AF448071
AF139301	AF247574	AF317074	AF448069
AF139313	AF216950	AF317073	AF448068
AF139325	AF247589	AF317079	AF448062
AF092504	AF247586	AF317076	AF448080
AF115268	AF247571	AF317077	AF506247
AF115269	AF247583	AF317632	AF506239
AF160256	AF247579	AF330253	AF506246
AF132779	AF247591	AF329393	AF506251
AF139297	AF260509	AF329392	AF506259
AF139309	AF260510	AF330252	AF506264
AF139321	AF268004	AF330246	AF506245
AF180967	AF268016	AF329391	AF506243
AF139298	AF268010	AF330247	AF506258
AF139310	AF268014	AF330254	AF506244
AF139322	AF268022	AF330251	AF506257
AF027655	AF268007	AF317071	AF506263
AF216945	AF268015	AF317630	AF506242
AF216954	AF268006	AF330250	AF506255
AF216944	AF268019	AF216948	AF506240
AF218377	AF268018	AF330249	AF506252
AF216951	AF268009	AF247580	AF506265
AF216947	AF268021	AF268011	AF506248
AF216953	AF268008	AF268023	AF506261
AF218371	AF268005	AF382136	AF448061
AF216955	AF268017	AF382121	AF516727
AF216952	AF247581	AF382128	AF516739
AF218373	AF247582	AF382140	AF516733
AF218372	AF247594	AF382117	AF516725
AF062638	AF139296	AF284803	AF516745
AF076162	AF139308	AF400152	AF516726
AF216949	AF139320	AF407696	AF516730
AF245618	AF268012	AF407731	AF516737
AF218368	AF268024	AF330248	AF516724
AF247573	AF268013	AF445673	AF516738
AF247585	AF268237	AF445667	AF516732
AF247575	AF311219	AF445691	AF516742
AF247587	AF311218	AF448063	AF516736
AF247576	AF311292	AF448060	AF516729
AF247572	AF311220	AF448072	AF516744
AF247578	AF311293	AF448064	AF516741

Table S1. *Cont.*

Accession number	Accession number	Accession number	Accession number
AF516728	AJ133172	AJ630424	AJ635435
AF516735	AJ133184	AJ630432	AJ639896
AF516731	AJ133153	AJ630413	AJ639891
AF516740	AJ133165	AJ630419	AJ639897
AF516743	AJ133177	AJ630436	AJ639901
AF539812	AJ245457	AJ630444	AJ639898
AF506237	AJ289785	AJ630425	AJ639899
AF506262	AJ293106	AJ630431	AJ781135
AF516734	AJ293118	AJ630418	AJ781147
AF516746	AJ293130	AJ630448	AJ781141
AFJC01000029	AJ293112	AJ630417	AJ781134
AGIZ01000009	AJ293105	AJ630437	AJ781146
AGSF01001460	AJ293124	AJ630409	AJ781137
AJ006282	AJ293102	AJ630416	AJ781149
AJ133159	AJ293117	AJ630443	AJ781136
AJ133154	AJ293108	AJ630430	AJ781139
AJ133171	AJ293114	AJ630411	AJ781148
AJ133166	AJ293129	AJ630415	AJ781151
AJ133183	AJ293122	AJ630646	AJ781132
AJ133178	AJ293120	AJ630429	AJ781138
AJ133173	AJ293103	AJ630449	AJ781144
AJ133185	AJ293126	AJ630421	AJ781140
AJ006281	AJ293104	AJ630442	AJ781133
AJ133155	AJ293115	AJ630434	AJ781145
AJ133167	AJ293116	AJ630423	AJ781131
AJ133179	AJ293127	AJ630427	AJ781143
AJ132250	AJ293128	AJ630441	AM084273
AJ132251	AJ293113	AJ630433	AM230685
AJ133156	AJ293125	AJ630440	AM230690
AJ133168	AJ293111	AJ630446	AM230698
AJ133180	AJ293107	AJ630435	AM230687
AJ133157	AJ293123	AJ630439	AM230700
AJ133151	AJ293119	AJ630453	AM230681
AJ133174	AJ293131	AJ630445	AM230693
AJ133169	AJ293109	AJ630414	AM230684
AJ133181	AJ293121	AJ630447	AM230697
AJ133158	AJ344563	AJ630426	AM230679
AJ133175	AJ347056	AJ630438	AM230680
AJ133170	AJ544078	AJ635431	AM230691
AJ133182	AJ582102	AJ635430	AM230692
AJ224447	AJ630410	AJ635432	AM230682
AJ133152	AJ630422	AJ635433	AM230694
AJ133164	AJ630408	AJ635429	AM230686
AJ133176	AJ630412	AJ635434	AM230699
AJ133160	AJ630420	AJ639900	AM236076

Table S1. *Cont.*

Accession number	Accession number	Accession number	Accession number
AM230688	AM710375	AP008231	AY151236
AM230701	AM710357	AY038035	AY151248
AM230683	AM710368	AP009552	AY125385
AM230695	AM710361	AP009552	AY151239
AM230689	AM710369	AY033310	AY151729
AM259254	AM710380	AY038032	AY151251
AM259253	AM710373	AY112695	AY151725
AM259221	AM710381	AY038034	AY151237
AM259804	AM711531	AY125364	AY151241
AM259247	AM710346	AY125376	AY151232
AM259271	AM711536	AY125360	AY151249
AM259815	AM710358	AY125372	AY151244
AM259222	AM710347	AY125362	AY151727
AM259246	AM711553	AY125384	AY151735
AM259251	AM710350	AY125371	AY151730
AM259270	AM710370	AY125383	AY151236
AM259252	AM710359	AY125370	AY151248
AM259800	AM710362	AY125382	AY125366
AM259860	AM710382	AY125374	AY125378
AM259793	AM710371	AY125386	AY135675
AM259813	AM710374	AY125365	AY125368
AM259861	AM710383	AY125377	AY125380
AM259798	AM711526	AY125369	AY125363
AM709628	AM711527	AY125381	AY125375
AM709629	AM711539	AY125367	AY151240
AM709626	AM711542	AY151243	AY151726
AM709627	AM711554	AY125379	AY151234
AM710352	AM711535	AY151238	AY151246
AM710364	AM710353	AY151250	AY151732
AM710376	AM710365	AY151724	AY168749
AM710348	AM710377	AY125361	AY172809
AM710343	AM710342	AY125373	AY172821
AM710360	AM710354	AY125385	AY170472
AM710355	AM710366	AY151239	AY151233
AM710372	AM710378	AY151729	AY151245
AM710367	AM711545	AY151251	AY172806
AM710379	AM711546	AY151725	AY172818
AM710351	AM747367	AY151237	AY172830
AM711528	AM746687	AY151241	AY172807
AM710344	AM940218	AY151232	AY172811
AM710363	AM940219	AY151249	AY172819
AM710345	AP008231	AY151244	AY172823
AM711540	AY033297	AY151727	AY172801
AM710356	AY033307	AY151735	AY172833
AM710349	AY033308	AY151730	AY172835

Table S1. *Cont.*

Accession number	Accession number	Accession number	Accession number
AY172808	AY328614	AY575925	AY701549
AY172813	AY328573	AY575930	AY701567
AY172802	AY328898	AY575924	AY701561
AY172820	AY328900	AY575931	AY701547
AY172825	AY328897	AY575928	AY701559
AY172814	AY328899	AY575927	AY701542
AY172832	AY328896	AY575923	AY701571
AY172837	AY344431	AY663904	AY701551
AY172826	AY354194	AY672730	AY701554
AY151235	AY439283	AY672713	AY701563
AY151247	AY439281	AY672723	AY701566
AY151721	AY439282	AY672729	AY742449
AY151733	AY493573	AY672732	AY742453
AY183114	AY493577	AY672725	AY742451
AY183115	AY493611	AY672715	AY752088
AY151242	AY493583	AY672727	AY763116
AY151728	AY493623	AY672733	AY763117
AY172810	AY493595	AY672728	AY862014
AY172822	AY493607	AY672718	AY862011
AY172834	AY493576	AY672721	AY881622
AY172804	AY493578	AY672734	AY907760
AY172816	AY493590	AY672720	AY930443
AY172828	AY493612	AY672731	AY942769
AY172803	AY493572	AY700634	AY945298
AY172815	AY493581	AY699989	AY943947
AY172805	AY493593	AY701541	AY945295
AY172817	AY493574	AY701545	AY945292
AY172829	AY493580	AY701553	AY945300
AY196083	AY493605	AY701565	AY945296
AY196082	AY493592	AY701546	AY945293
AY196084	AY493579	AY701569	AY946244
AY196086	AY493984	AY700638	AY945294
AY172800	AY493604	AY701570	BD061303
AY172812	AY493582	AY701544	BX548174
AY172824	AY493591	AY701556	BX548175
AY172836	AY493594	AY701568	BX569694
AY196088	AY493603	AY701552	BX569694
AY196085	AY493606	AY701548	BX548175
AY196087	AY493615	AY701564	CP000097
AY224194	AY536229	AY701550	CP000095
AY224199	AY566855	AY701560	CP000110
AY224198	AY575929	AY701562	CP000100
AY218833	AY575932	AY701572	CP000100
AY224200	AY575933	AY701543	CP000110
AY328578	AY575926	AY701555	CP000097

Table S1. *Cont.*

Accession number	Accession number	Accession number	Accession number
CP000111	DQ070784	DQ185214	DQ279771
CP000435	DQ071047	DQ185220	DQ279767
CP000554	DQ071169	DQ185227	DQ279772
CP000554	DQ071032	DQ185216	DQ279770
CP000553	DQ071062	DQ185206	DQ279768
CP000435	DQ071158	DQ185236	DQ279769
CP000551	DQ088155	DQ185247	DQ300610
CP000815	DQ124252	DQ185225	DQ300617
CP000552	DQ129320	DQ185226	DQ300603
CP000576	DQ131173	DQ185232	DQ300601
CP000825	DQ181682	DQ185239	DQ300621
CP000815	DQ181673	DQ185228	DQ300625
CP000878	DQ181685	DQ185205	DQ330775
CP001037	DQ181742	DQ185218	DQ330753
CP001037	DQ181680	DQ185243	DQ363254
CP001037	DQ181669	DQ185248	DQ366018
CP001037	DQ181716	DQ185259	DQ366714
CT978603	DQ181681	DQ185237	DQ366719
CT971583	DQ181740	DQ185238	DQ378254
CT971583	DQ181674	DQ185244	DQ393285
D89033	DQ181686	DQ185251	DQ393284
D89034	DQ181678	DQ185217	DQ393281
D89036	DQ181683	DQ185230	DQ393279
D89032	DQ181675	DQ185255	DQ393283
D88288	DQ181684	DQ185249	DQ393282
D89031	DQ181687	DQ185250	DQ393280
DQ009327	DQ181676	DQ185209	DQ396291
DQ009353	DQ181707	DQ185256	DQ431002
DQ009324	DQ181677	DQ185252	DQ431005
DQ009326	DQ181688	DQ185229	DQ431004
DQ009318	DQ181714	DQ185222	DQ431003
DQ009357	DQ181715	DQ185221	DQ463712
DQ009354	DQ181701	DQ185241	DQ471444
DQ009320	DQ185210	DQ185254	DQ471442
DQ009358	DQ185211	DQ185234	DQ471447
DQ009317	DQ185203	DQ185233	DQ471446
DQ009356	DQ185207	DQ185253	DQ490036
DQ009322	DQ185212	DQ185246	DQ493873
DQ009321	DQ185223	DQ185245	DQ493874
DQ009359	DQ185215	DQ185258	DQ521499
DQ009355	DQ185204	DQ185257	DQ521503
DQ023200	DQ185219	DQ269094	DQ532167
DQ023199	DQ185224	DQ275602	DQ532230
DQ009323	DQ185235	DQ275599	DQ532224
DQ070786	DQ185213	DQ279773	DQ648029

Table S1. *Cont.*

Accession number	Accession number	Accession number	Accession number
DQ648028	EF126264	EF174218	EF432314
DQ648027	EF126269	EF174227	EF432313
DQ648030	EF126240	EF174221	EF432320
DQ648026	EF126276	EF174230	EF432318
DQ786172	EF126226	EF174233	EF432312
DQ786171	EF126281	EF174210	EF438248
DQ786170	EF126270	EF174222	EF451600
DQ786169	EF126242	EF174211	EF471456
DQ786166	EF126282	EF174223	EF515909
DQ786173	EF126268	EF174205	EF516167
DQ914863	EF126267	EF174217	EF516076
EF032660	EF126280	EF174229	EF516229
EF032785	EF126236	EF174202	EF516408
EF032779	EF126275	EF174214	EF516746
EF032781	EF126273	EF174226	EF520519
EF032782	EF126274	EF175744	EF522223
EF032789	EF159841	EF205458	EF522272
EF032663	EF159853	EF205461	EF522247
EF032786	EF159840	EF205482	EF522296
EF076234	EF159843	EF205455	EF522259
EF076240	EF159868	EF205546	EF522222
EF088337	EF159845	EF222475	EF522264
EF088333	EF159835	EF429500	EF522246
EF088334	EF159872	EF429512	EF522295
EF088332	EF159922	EF429524	EF522225
EF088341	EF159884	EF429511	EF522288
EF088339	EF174201	EF429523	EF522251
EF111085	EF174204	EF429510	EF522233
EF121241	EF174213	EF429504	EF522324
EF123580	EF174216	EF429502	EF522273
EF123579	EF174207	EF429516	EF522310
EF126222	EF174200	EF429514	EF522318
EF126247	EF174225	EF429526	EF522269
EF126237	EF174228	EF429527	EF522267
EF126215	EF174219	EF429525	EF522345
EF126254	EF174212	EF429509	EF522303
EF126221	EF174208	EF429506	EF522349
EF126223	EF174231	EF429521	EF529482
EF126239	EF174224	EF429520	EF529483
EF126233	EF174203	EF429519	EF529489
EF126216	EF174220	EF429518	EF529484
EF126278	EF174206	EF432316	EF529477
EF126228	EF174215	EF432317	EF529485
EF126218	EF174232	EF432311	EF536023
EF126248	EF174209	EF432319	EF536021

Table S1. *Cont.*

Accession number	Accession number	Accession number	Accession number
EF536020	EF568908	EF572237	EF572845
EF536024	EF568889	EF572303	EF572897
EF536022	EF568914	EF572285	EF572904
EF545607	EF568891	EF572316	EF572901
EF547194	EF568905	EF572309	EF572909
EF547190	EF568871	EF572361	EF572916
EF547191	EF568920	EF572416	EF572920
EF547195	EF568901	EF572381	EF572940
EF547192	EF568903	EF572411	EF572926
EF547196	EF568875	EF572433	EF572983
EF547193	EF568917	EF572437	EF572951
EF568873	EF568883	EF572405	EF572995
EF568885	EF568913	EF572408	EF572976
EF568897	EF568915	EF572472	EF572998
EF568909	EF568887	EF572493	EF572999
EF568921	EF568895	EF572475	EF573099
EF564663	EF568899	EF572517	EF573056
EF568868	EF568911	EF572529	EF573042
EF568874	EF568919	EF572584	EF573134
EF568870	EF571914	EF572511	EF573100
EF568892	EF571928	EF572558	EF573139
EF568882	EF571976	EF572610	EF573117
EF568904	EF571981	EF572618	EF573206
EF568876	EF572047	EF572605	EF573138
EF568872	EF572039	EF572634	EF573290
EF568886	EF572077	EF572541	EF573234
EF568878	EF572103	EF572694	EF573269
EF568894	EF572010	EF572566	EF573270
EF568869	EF572087	EF572693	EF573435
EF568916	EF572224	EF572590	EF573338
EF568888	EF572132	EF572602	EF573172
EF568884	EF572043	EF572755	EF573531
EF568898	EF572055	EF572620	EF573191
EF568890	EF572068	EF572680	EF573251
EF568906	EF572050	EF572686	EF573342
EF568881	EF572192	EF572724	EF573288
EF568900	EF572111	EF572716	EF573300
EF568896	EF572133	EF572718	EF573439
EF568877	EF572247	EF572768	EF573370
EF568910	EF572170	EF572747	EF573497
EF568902	EF572167	EF572800	EF573533
EF568918	EF572210	EF572818	EF573489
EF568879	EF572233	EF572852	EF573565
EF568893	EF572239	EF572837	EF573558
EF568912	EF572243	EF572849	EF573577

Table S1. *Cont.*

Accession number	Accession number	Accession number	Accession number
EF573697	EF573965	EF574031	EF574160
EF573537	EF573977	EF574010	EF574182
EF573663	EF573716	EF574043	EF574203
EF573576	EF574056	EF573993	EF574262
EF573757	EF574057	EF574036	EF574300
EF573708	EF573739	EF574095	EF574213
EF573769	EF573989	EF574038	EF574271
EF573586	EF573751	EF574048	EF574194
EF573732	EF573740	EF574144	EF574215
EF573699	EF573729	EF574116	EF574274
EF573676	EF574081	EF574200	EF574225
EF573695	EF573786	EF574017	EF574237
EF573805	EF573787	EF574030	EF574229
EF573737	EF573776	EF574070	EF574295
EF573700	EF573822	EF574128	EF574239
EF573719	EF573812	EF574168	EF574298
EF573735	EF574152	EF574041	EF574336
EF573731	EF573813	EF574180	EF574219
EF573780	EF573826	EF574094	EF574251
EF573658	EF574164	EF574151	EF574310
EF573668	EF573858	EF574154	EF574348
EF573759	EF573902	EF574106	EF574405
EF573670	EF573916	EF574098	EF574322
EF573678	EF573935	EF574087	EF574265
EF573785	EF573873	EF574118	EF574331
EF573703	EF573885	EF574187	EF574232
EF573706	EF573907	EF574102	EF574372
EF573714	EF573940	EF574141	EF574277
EF573726	EF573959	EF574134	EF574255
EF573821	EF573919	EF574202	EF574346
EF573784	EF574037	EF574174	EF574289
EF573843	EF573971	EF574211	EF574453
EF573855	EF573950	EF574296	EF574302
EF573949	EF573922	EF574126	EF574333
EF573832	EF573943	EF574149	EF574292
EF573900	EF573974	EF574167	EF574366
EF573961	EF573933	EF574226	EF574304
EF573879	EF573955	EF574177	EF574445
EF573973	EF573976	EF574169	EF574357
EF573924	EF574035	EF574235	EF574431
EF573905	EF574007	EF574320	EF574403
EF573972	EF573967	EF574173	EF574395
EF573984	EF574047	EF574222	EF574476
EF573996	EF574085	EF574344	EF574463

Table S1. *Cont.*

Accession number	Accession number	Accession number	Accession number
EF574475	EF574599	EF574756	EF575063
EF574621	EF574550	EF574754	EF574959
EF574480	EF574564	EF574932	EF575044
EF574511	EF574562	EF574787	EF575136
EF574633	EF574557	EF574724	EF575018
EF574551	EF574558	EF574749	EF574971
EF574572	EF574623	EF574770	EF575056
EF574608	EF574569	EF574815	EF575002
EF574693	EF574570	EF574768	EF575068
EF574316	EF574685	EF574853	EF575160
EF574644	EF574602	EF574771	EF574995
EF574338	EF574614	EF574761	EF575054
EF574729	EF574659	EF574782	EF575078
EF574349	EF574580	EF574827	EF575116
EF574656	EF574606	EF574794	EF575090
EF574350	EF574617	EF574823	EF575128
EF574361	EF574721	EF574760	EF575074
EF574668	EF574813	EF574785	EF575174
EF574377	EF574667	EF574806	EF575152
EF574777	EF574616	EF574889	EF575164
EF574393	EF574642	EF574919	EF575259
EF574410	EF574662	EF574797	EF575138
EF574406	EF574658	EF574818	EF575091
EF574425	EF574719	EF574814	EF575203
EF574446	EF574849	EF574842	EF575307
EF574412	EF574665	EF574838	EF575282
EF574454	EF574769	EF574871	EF574939
EF574424	EF574687	EF574854	EF574975
EF574449	EF574800	EF575028	EF574937
EF574450	EF574678	EF574910	EF575047
EF574466	EF574727	EF574874	EF574940
EF574471	EF574689	EF575052	EF574949
EF574461	EF574710	EF574918	EF574953
EF574460	EF574755	EF574891	EF574974
EF574502	EF574708	EF575003	EF574976
EF574516	EF574739	EF574946	EF575000
EF574518	EF574711	EF575076	EF575010
EF574514	EF574722	EF574909	EF575012
EF574547	EF574805	EF575088	EF575021
EF574509	EF574763	EF574914	EF575131
EF574526	EF574920	EF574923	EF575024
EF574559	EF574712	EF574892	EF575025
EF574496	EF574860	EF574982	EF575034
EF574587	EF574737	EF575051	EF575037
EF574538	EF574758	EF574978	EF574993

Table S1. *Cont.*

Accession number	Accession number	Accession number	Accession number
EF575048	EF580958	EU022717	EU078539
EF575167	EF580987	EU022707	EU078538
EF575082	EF583861	EU022742	EU078486
EF575120	EF583863	EU022729	EU078498
EF575077	EF583853	EU022741	EU078492
EF575133	EF583854	EU022731	EU078504
EF575145	EF583860	EU022715	EU078522
EF575169	EF583859	EU022714	EU078534
EF575115	EF583858	EU022727	EU078484
EF575127	EF630354	EU022710	EU078540
EF575196	EF632880	EU022726	EU078496
EF575146	EF654079	EU022739	EU078489
EF575149	EF654066	EU022725	EU078482
EF575177	EF654078	EU022711	EU078487
EF575173	EF654081	EU022722	EU078499
EF575232	EF654071	EU022738	EU078494
EF575185	EF654077	EU022737	EU078506
EF575243	EF654068	EU022712	EU078518
EF575197	EF654082	EU022723	EU078520
EF575200	EF654072	EU022734	EU078501
EF575251	EF654064	EU022724	EU078535
EF575264	EF654063	EU022735	EU078530
EF575263	EF654084	EU022733	EU078500
EF575280	EF654076	EU022716	EU078532
EF575276	EF654062	EU022736	EU078495
EF575277	EF654074	EU022728	EU078547
EF575226	EF654073	EU022708	EU078542
EF575275	EF667962	EU022740	EU078544
EF575236	EF683072	EU022732	EU078507
EF575291	EU000446	EU037931	EU078533
EF575238	EU000444	EU076459	EU078541
EF575287	EU009154	EU076457	EU078524
EF575248	EU009149	EU076458	EU078519
EF575318	EU009152	EU078485	EU078537
EF575304	EU010175	EU078497	EU078536
EF575315	EU010221	EU078491	EU078531
EF575262	EU010199	EU078490	EU078548
EF575311	EU010216	EU078503	EU078543
EF575274	EU010223	EU078521	EU091555
EF575296	EU010179	EU078502	EU091558
EF575309	EU010210	EU078493	EU091546
EF575305	EU015871	EU078488	EU091541
EF575310	EU022713	EU078527	EU151901
EF575321	EU022709	EU078505	EU151902
EF575322	EU022730	EU078526	EU157985

Table S1. *Cont.*

Accession number	Accession number	Accession number	Accession number
EU157997	EU237300	EU237414	EU394578
EU157979	EU237326	EU237426	EU427543
EU157991	EU237304	EU237404	EU439566
EU157983	EU237295	EU237428	EU528248
EU157976	EU237337	EU237452	EU541974
EU157995	EU237309	EU237464	EU541972
EU157986	EU237325	EU249939	EU541973
EU157998	EU237317	EU251112	EU541970
EU157981	EU237274	EU255712	EU541971
EU157984	EU237315	EU255703	EU552060
EU157993	EU237329	EU255715	EU552058
EU157996	EU237286	EU255713	EU552064
EU157975	EU237395	EU255708	EU552066
EU157987	EU237407	EU255720	EU552069
EU157999	EU237399	EU255710	EU552056
EU157980	EU237331	EU255722	EU552055
EU157992	EU237323	EU255705	EU552068
EU157982	EU237397	EU255717	EU552065
EU157994	EU237321	EU255707	EU552067
EU157978	EU237411	EU255719	EU552063
EU157977	EU237423	EU255711	EU552062
EU157990	EU237443	EU255718	EU552059
EU157989	EU237433	EU255704	EU552057
EU178142	EU237455	EU255709	EU552061
EU178144	EU237447	EU255716	EU552070
EU178143	EU237398	EU255721	EU586733
EU183352	EU237412	EU255702	EU592410
EU188151	EU237467	EU255714	EU636199
EU236282	EU237410	EU259800	EU703165
EU237269	EU237424	EU282430	EU703155
EU237281	EU237427	EU282432	EU703193
EU237318	EU237413	EU282431	EU703191
EU237275	EU237429	EU340178	EU703160
EU237330	EU237444	EU340181	EU703175
EU237267	EU237439	EU340159	EU703185
EU237299	EU237394	EU340174	EU703252
EU237289	EU237441	EU340164	EU703250
EU237276	EU237406	EU340161	EU703182
EU237268	EU237437	EU340230	EU703154
EU237301	EU237418	EU340209	EU703201
EU237266	EU237449	EU340177	EU703265
EU237288	EU237458	EU340168	EU703264
EU237314	EU237465	EU340187	EU703190
EU237328	EU237472	EU340180	EU703228
EU237285	EU237470	EU340217	EU703248

Table S1. *Cont.*

Accession number	Accession number	Accession number	Accession number
EU703244	EU703496	EU753642	EU802415
EU703262	EU703468	EU753638	EU802483
EU703209	EU703425	EU753636	EU802427
EU703258	EU703481	EU753637	EU802507
EU703249	EU703487	EU753622	EU802615
EU703334	EU703491	EU753628	EU802544
EU703255	EU703473	EU753633	EU802557
EU703263	EU703464	EU753629	EU802559
EU703173	EU703483	EU753640	EU802564
EU703253	EU703477	EU753641	EU802651
EU703283	EU703467	EU780160	EU802671
EU703202	EU703488	EU780158	EU802573
EU703329	EU703482	EU780161	EU802594
EU703345	EU703497	EU780159	EU802604
EU703342	EU703184	EU780156	EU802619
EU703326	EU703200	EU802245	EU802724
EU703362	EU703243	EU802261	EU802593
EU703170	EU703257	EU802273	EU802640
EU703358	EU703328	EU802296	EU802611
EU703246	EU703357	EU802308	EU802589
EU703346	EU703247	EU802344	EU802630
EU703254	EU703472	EU802314	EU802789
EU703355	EU703360	EU802369	EU802810
EU703260	EU703462	EU802389	EU802716
EU703363	EU704749	EU802277	EU802729
EU703199	EU704866	EU802442	EU802741
EU703368	EU704819	EU802250	EU802851
EU703364	EU704992	EU802300	EU802738
EU703366	EU705079	EU802322	EU802808
EU703256	EU705186	EU802298	EU802799
EU703312	EU744336	EU802337	EU802907
EU703325	EU753632	EU802531	EU802836
EU703463	EU753644	EU802407	EU802862
EU703337	EU753623	EU802405	EU802873
EU703476	EU753634	EU802413	EU802896
EU703359	EU753627	EU802354	EU802964
EU703437	EU753646	EU802542	EU802826
EU703493	EU753639	EU802535	EU803026
EU703367	EU753624	EU802431	EU802843
EU703327	EU753635	EU802588	EU803051
EU703465	EU753631	EU802432	EU802818
EU703478	EU753630	EU802579	EU803063
EU703495	EU753626	EU802472	EU802866
EU703369	EU753625	EU802613	EU802879
EU703479	EU753643	EU802635	EU802900

Table S1. *Cont.*

Accession number	Accession number	Accession number	Accession number
EU803078	EU804102	EU804481	EU804783
EU802928	EU804117	EU804526	EU804810
EU802950	EU804137	EU804576	EU804817
EU803073	EU804264	EU804588	EU804821
EU802963	EU804151	EU804607	EU804955
EU802979	EU804142	EU804561	EU804916
EU803097	EU804128	EU804533	EU804913
EU803128	EU804250	EU804571	EU804902
EU803125	EU804160	EU804578	EU804896
EU803035	EU804164	EU804582	EU804898
EU803053	EU804209	EU804596	EU804952
EU802993	EU804283	EU804654	EU804918
EU803060	EU804215	EU804628	EU804939
EU803055	EU804226	EU804696	EU804946
EU803057	EU804253	EU804675	EU805036
EU803202	EU804323	EU804676	EU805074
EU803173	EU804374	EU804756	EU804965
EU803157	EU804275	EU804716	EU804942
EU803076	EU804268	EU804740	EU804966
EU803185	EU804261	EU804852	EU805090
EU803087	EU804305	EU804825	EU805019
EU803025	EU804307	EU804859	EU805115
EU803201	EU804300	EU804857	EU805007
EU803071	EU804335	EU804870	EU805069
EU803192	EU804280	EU804882	EU805141
EU803129	EU804344	EU804897	EU805138
EU803141	EU804343	EU805001	EU805062
EU803110	EU804329	EU805026	EU805042
EU803159	EU804378	EU805063	EU805067
EU803189	EU804361	EU804531	EU805162
EU803122	EU804349	EU804591	EU805054
EU803163	EU804394	EU804635	EU805056
EU803773	EU804388	EU804669	EU805082
EU804045	EU804432	EU804624	EU805107
EU804069	EU804385	EU804633	EU805198
EU804081	EU804464	EU804646	EU805096
EU804082	EU804463	EU804658	EU805214
EU804095	EU804466	EU804668	EU805131
EU804103	EU804479	EU804731	EU805125
EU804134	EU804470	EU804725	EU805123
EU804111	EU804473	EU804820	EU805111
EU804173	EU804512	EU804739	EU805133
EU804145	EU804552	EU804748	EU805146
EU804157	EU804522	EU804773	EU805168
EU804186	EU804509	EU804792	EU805180

Table S1. *Cont.*

Accession number	Accession number	Accession number	Accession number
EU805218	FJ184427	FJ184389	FJ424556
EU805286	FJ184431	FJ184401	FJ424571
EU805221	FJ184440	FJ184413	FJ424563
EU805266	FJ184396	FJ184425	FJ424568
EU805207	FJ184061	FJ184438	FJ424575
EU805233	FJ184058	FJ192908	FJ424581
EU805346	FJ184408	FJ203301	FJ424562
EU805158	FJ184420	FJ230782	FJ424561
EU805260	FJ184387	FJ230783	FJ424574
EU805194	FJ184432	FJ230790	FJ424573
EU805341	FJ184399	FJ230802	FJ424554
EU805211	FJ184393	FJ230815	FJ424564
EU805313	FJ184433	FJ230787	FJ424566
EU805378	FJ184411	FJ230792	FJ424557
EU805245	FJ184385	FJ230814	FJ424576
EU805267	FJ184405	FJ230791	FJ424565
EU805392	FJ184423	FJ230784	FJ424579
EU805309	FJ184390	FJ230796	FJ424569
EU805299	FJ184417	FJ234884	FJ424558
EU805304	FJ184436	FJ234882	FJ424577
EU805328	FJ184402	FJ234898	FJ424578
EU805367	FJ184386	FJ234885	FJ424570
EU805372	FJ184429	FJ234895	FJ424555
EU805368	FJ184397	FJ234897	FJ424567
EU815063	FJ184394	FJ234891	FJ424560
EU815326	FJ184414	FJ234888	FJ424580
EU817117	FJ184398	FJ381981	FJ425596
EU817104	FJ184442	FJ381993	FJ434216
EU861839	FJ184409	FJ381980	FJ434249
EU861854	FJ184406	FJ382036	FJ434247
FB705429	FJ184426	FJ381976	FJ434248
FJ159128	FJ184410	FJ381997	FJ434223
FJ184059	FJ184421	FJ382023	FJ434250
FJ184388	FJ184418	FJ381978	FJ437751
FJ184400	FJ184439	FJ382028	FJ437767
FJ184412	FJ184422	FJ382022	FJ437750
FJ184391	FJ184434	FJ382002	FJ437819
FJ184395	FJ184430	FJ382043	FJ437825
FJ184424	FJ184435	FJ382039	FJ437887
FJ184403	FJ184443	FJ382047	FJ437897
FJ184407	FJ184392	FJ382194	FJ444635
FJ184437	FJ184404	FJ382111	FJ456919
FJ184415	FJ184416	FJ382144	FJ461749
FJ184419	FJ184428	FJ382618	FJ461750
FJ184060	FJ184441	FJ424559	FJ461751

Table S1. *Cont.*

Accession number	Accession number	Accession number	Accession number
FJ484406	FJ545514	FJ661006	FJ790640
FJ484408	FJ546716	FJ661013	FJ790611
FJ484388	FJ546713	FJ661018	FJ790623
FJ484368	FJ562137	FJ661016	FJ790554
FJ484830	FJ592872	FJ661011	FJ790617
FJ484842	FJ592871	FJ763773	FJ790635
FJ484873	FJ592873	FJ763785	FJ798612
FJ484823	FJ592868	FJ763768	FJ815296
FJ484833	FJ592874	FJ763766	FJ815308
FJ484866	FJ595684	FJ763780	FJ815302
FJ484876	FJ595687	FJ763778	FJ815314
FJ484891	FJ595696	FJ763772	FJ815301
FJ484881	FJ595692	FJ763765	FJ815294
FJ484819	FJ595686	FJ763784	FJ815313
FJ484841	FJ595694	FJ763777	FJ815298
FJ484834	FJ595698	FJ763776	FJ815306
FJ484849	FJ595691	FJ763788	FJ815310
FJ484867	FJ595616	FJ763775	FJ815318
FJ484864	FJ595688	FJ763769	FJ815303
FJ484857	FJ595689	FJ763787	FJ815315
FJ484850	FJ595693	FJ763781	FJ815295
FJ484885	FJ595685	FJ763771	FJ815304
FJ497717	FJ595697	FJ763770	FJ815320
FJ497730	FJ595690	FJ763767	FJ815307
FJ497736	FJ595695	FJ763783	FJ815316
FJ497720	FJ612266	FJ763782	FJ815319
FJ497718	FJ612304	FJ763779	FJ815321
FJ497746	FJ612215	FJ763774	FJ815291
FJ497722	FJ612211	FJ763789	FJ815293
FJ497737	FJ612417	FJ763786	FJ815299
FJ497739	FJ612247	FJ790541	FJ815305
FJ497729	FJ612296	FJ790563	FJ815297
FJ497719	FJ612358	FJ788926	FJ815311
FJ497733	FJ612386	FJ790616	FJ815300
FJ497724	FJ612410	FJ790621	FJ815317
FJ497741	FJ638532	FJ790602	FJ815309
FJ497725	FJ661007	FJ790634	FJ815312
FJ497745	FJ661019	FJ790644	FJ820438
FJ497743	FJ661010	FJ790555	FJ820420
FJ497721	FJ661015	FJ790610	FJ820461
FJ497744	FJ661014	FJ790620	FJ826625
FJ497716	FJ661017	FJ790618	FJ826622
FJ497735	FJ661021	FJ790636	FJ826623
FJ497747	FJ661008	FJ790642	FJ826624
FJ517069	FJ661012	FJ790628	FJ830577

Table S1. *Cont.*

Accession number	Accession number	Accession number	Accession number
FJ828955	FJ849099	FJ890633	FJ902666
FJ830639	FJ849176	FJ891015	FJ902586
FJ830573	FJ849198	FJ890630	FJ902597
FJ830578	FJ849139	FJ890615	FJ902620
FJ830576	FJ849205	FJ890617	FJ902648
FJ830581	FJ849228	FJ890616	FJ902609
FJ830574	FJ849166	FJ890634	FJ902664
FJ830579	FJ849240	FJ890624	FJ902660
FJ830570	FJ849160	FJ890631	FJ933259
FJ830580	FJ849281	FJ890622	FJ937849
FJ830571	FJ849293	FJ890623	FJ937843
FJ830572	FJ849289	FJ890629	FJ937846
FJ839352	FJ849218	FJ890620	FJ937858
FJ839360	FJ849230	FJ890990	FJ937856
FJ847376	FJ849237	FJ890627	FJ937848
FJ847393	FJ849244	FJ891051	FJ937844
FJ847390	FJ849247	FJ891012	FJ937860
FJ847377	FJ849271	FJ891032	FJ937842
FJ847389	FJ849308	FJ891036	FJ937852
FJ847379	FJ849316	FJ891028	FJ937851
FJ847394	FJ866615	FJ901434	FJ937857
FJ847382	FJ866617	FJ901813	FJ946524
FJ847384	FJ866612	FJ902594	FJ973577
FJ847387	FJ866616	FJ902579	FJ982323
FJ847396	FJ866618	FJ902584	FJ999597
FJ847378	FJ866614	FJ902634	FJ999607
FJ847395	FJ885804	FJ902577	FJ999609
FJ847374	FJ885895	FJ902607	FJ999601
FJ847391	FJ885896	FJ902581	FJ999594
FJ847380	FJ885782	FJ902619	FJ999593
FJ847385	FJ885852	FJ902614	FJ999612
FJ847397	FJ885984	FJ902596	FM161347
FJ847375	FJ885965	FJ902653	FM177491
FJ847392	FJ886068	FJ902631	FM177502
FJ847383	FJ886676	FJ902616	FM177484
FJ849078	FJ890621	FJ902665	FM177478
FJ847388	FJ890628	FJ902636	FM177496
FJ847381	FJ890618	FJ902643	FM177490
FJ847386	FJ891009	FJ902590	FM177501
FJ847398	FJ890632	FJ902599	FM177476
FJ849213	FJ890625	FJ902649	FM177488
FJ849265	FJ890619	FJ902640	FM177499
FJ849122	FJ890626	FJ902659	FM177485
FJ849313	FJ890614	FJ902632	FM177497
FJ849211	FJ890995	FJ902654	FM177483

Table S1. *Cont.*

Accession number	Accession number	Accession number	Accession number
FM177495	FN691920	FR667519	FR667443
FM177477	FN691914	FR667351	FR667467
FM177489	FN691926	FR667363	FR667491
FM177492	FN811217	FR667411	FR667503
FM177474	FN811237	FR667470	FR667532
FM177475	FN811218	FR667547	FR684854
FM177487	FN813344	FR667439	FR686005
FM177494	FN811239	FR667451	FR744555
FM177504	FN869878	FR667436	FR744616
FM177486	FN869879	FR667505	FR798936
FM177498	FN869883	FR667517	FR798919
FM210757	FN869880	FR667540	FR798931
FM210758	FN984809	FR667472	FR798940
FM242086	FN984821	FR667399	FR798933
FM242088	FN984833	FR667331	FR798945
FM242087	FN984880	FR667342	FR798925
FM242085	FN984881	FR667354	FR798937
FM242083	FN984814	FR667457	FR798939
FM242396	FN984863	FR667366	FR798938
FM242084	FN984820	FR667391	FR798917
FM872829	FN984803	FR667523	FR798941
FM995186	FN984813	FR667546	FR798935
FM995187	FN869882	FR667426	FR798918
FM995188	FN869881	FR667473	FR798942
FN678905	FN984871	FR667497	FR798944
FN689797	FN984819	FR667293	FW306008
FN689798	FN984852	FR667367	GQ130146
FN691907	FN984842	FR667404	GQ130142
FN691913	FN984802	FR667486	GQ130145
FN691919	FN984825	FR667516	GQ130154
FN691925	FN984836	FR667359	GQ130148
FN691916	FN984801	FR667396	GQ130151
FN691912	FN984811	FR667520	GQ130150
FN691924	FN984873	FR667531	GQ130147
FN691909	FR667251	FR667352	GQ130144
FN691911	FR667299	FR667389	GQ130143
FN691921	FR667314	FR667424	GQ130156
FN691923	FR667326	FR667495	GQ130153
FN691905	FR667418	FR667236	GQ130155
FN691906	FR667456	FR667513	GQ130149
FN691918	FR667465	FR667334	GQ130152
FN691917	FR667510	FR667345	GQ131854
FN691910	FR667489	FR667369	GQ162223
FN691922	FR667501	FR667429	GQ162224
FN691908	FR667507	FR667488	GQ184184

Table S1. *Cont.*

Accession number	Accession number	Accession number	Accession number
GQ184185	GQ441286	GQ994997	GU061530
GQ206141	GQ441321	GQ995001	GU061523
GQ206140	GQ441288	GQ994995	GU061556
GQ250621	GQ441352	GQ995000	GU061539
GQ324966	GQ457311	GQ994998	GU061549
GQ324967	GQ457312	GU056112	GU061533
GQ324964	GQ457309	GU061400	GU061529
GQ324969	GQ457310	GU061393	GU061558
GQ324972	GQ472781	GU061395	GU061542
GQ324963	GQ487938	GU061405	GU061535
GQ324965	GQ496077	GU061398	GU061568
GQ324968	GQ496078	GU061391	GU061551
GQ324973	GQ496079	GU061401	GU061545
GQ324971	GQ496080	GU061397	GU061541
GQ340089	GQ496076	GU061403	GU061524
GQ340158	GQ848192	GU061392	GU061570
GQ347849	GQ859597	GU061396	GU061554
GQ347850	GQ859601	GU061394	GU061580
GQ347904	GQ859603	GU061404	GU061528
GQ348763	GQ859600	GU061399	GU061563
GQ348648	GQ859598	GU061472	GU061573
GQ351564	GQ859621	GU061455	GU061557
GQ351570	GQ859618	GU061474	GU061553
GQ351576	GQ859633	GU061458	GU061526
GQ351563	GQ859624	GU061484	GU061536
GQ351567	GQ859602	GU061477	GU061582
GQ351573	GQ859622	GU061461	GU061531
GQ351572	GQ859620	GU061457	GU061559
GQ351575	GQ859636	GU061482	GU061540
GQ351574	GQ859634	GU061475	GU061575
GQ351566	GQ859652	GU061456	GU061585
GQ351569	GQ859632	GU061485	GU061569
GQ351565	GQ859649	GU061464	GU061565
GQ351578	GQ859619	GU061487	GU061538
GQ351577	GQ859645	GU061476	GU061548
GQ351568	GQ859631	GU061522	GU061543
GQ351571	GQ859651	GU061532	GU061578
GQ379567	GQ859599	GU061525	GU061571
GQ396895	GQ859623	GU061478	GU061552
GQ396938	GQ859635	GU061534	GU061587
GQ397056	GQ984313	GU061544	GU061581
GQ397048	GQ995002	GU061527	GU061577
GQ397093	GQ994999	GU061537	GU061550
GQ422955	GQ995003	GU061490	GU061560
GQ422959	GQ994996	GU061546	GU061555

Table S1. *Cont.*

Accession number	Accession number	Accession number	Accession number
GU061583	GU061754	GU061999	GU062172
GU061564	GU061728	GU061997	GU062110
GU061572	GU061763	GU062011	GU062163
GU061567	GU061749	GU061995	GU062141
GU061576	GU061740	GU062007	GU062109
GU061626	GU061761	GU061993	GU062175
GU061579	GU061752	GU061929	GU062153
GU061633	GU061764	GU061996	GU062133
GU061588	GU061830	GU062107	GU062121
GU061628	GU061562	GU062105	GU062111
GU061638	GU061574	GU062008	GU062165
GU061624	GU061584	GU062119	GU062143
GU061645	GU061627	GU062117	GU062112
GU061650	GU061625	GU061998	GU062125
GU061652	GU061639	GU062130	GU062145
GU061662	GU061637	GU062128	GU062132
GU061634	GU061926	GU062115	GU062177
GU061642	GU061635	GU062010	GU062155
GU061643	GU061663	GU062142	GU062123
GU061629	GU061661	GU062140	GU062137
GU061654	GU061647	GU062126	GU062157
GU061655	GU061723	GU062000	GU062144
GU061658	GU061721	GU062152	GU062146
GU061666	GU061636	GU062138	GU062149
GU061632	GU061733	GU062002	GU062169
GU061667	GU061747	GU062012	GU062156
GU061653	GU061648	GU062164	GU062158
GU061670	GU061745	GU062150	GU062179
GU061644	GU061731	GU061991	GU062147
GU061665	GU061759	GU062176	GU062161
GU061656	GU061660	GU062162	GU062168
GU061722	GU061757	GU062003	GU062159
GU061668	GU061743	GU062004	GU062173
GU061724	GU061669	GU062174	GU062171
GU061734	GU061755	GU062113	GU062468
GU061736	GU061729	GU062124	GU062166
GU061746	GU061732	GU062116	GU062178
GU061748	GU061741	GU062136	GU062469
GU061758	GU061744	GU062127	GU118171
GU061730	GU061753	GU062106	GU118278
GU061739	GU061756	GU062139	GU118332
GU061725	GU061726	GU062118	GU118385
GU061742	GU061738	GU062160	GU118310
GU061751	GU061750	GU062129	GU118371
GU061737	GU061927	GU062108	GU118398

Table S1. *Cont.*

Accession number	Accession number	Accession number	Accession number
GU118355	GU119217	GU119406	GU119390
GU118400	GU119250	GU119448	GU119392
GU118413	GU119255	GU119371	GU119410
GU118390	GU119292	GU119385	GU119413
GU118451	GU119215	GU119266	GU119422
GU118478	GU119283	GU119269	GU119491
GU118483	GU119267	GU119405	GU119449
GU118508	GU119306	GU119460	GU119440
GU118444	GU119227	GU119260	GU119462
GU118503	GU119241	GU119397	GU119464
GU118501	GU119261	GU119278	GU119482
GU118486	GU119295	GU119430	GU119474
GU118504	GU119274	GU119395	GU197632
GU118510	GU119239	GU119409	GU197644
GU118651	GU119291	GU119442	GU197656
GU118858	GU119336	GU119284	GU197631
GU118939	GU119251	GU119305	GU197643
GU119192	GU119265	GU119454	GU197655
GU119184	GU119285	GU119419	GU197622
GU119196	GU119277	GU119314	GU197634
GU119187	GU119297	GU119339	GU197629
GU119185	GU119289	GU119486	GU197646
GU119208	GU119322	GU119466	GU197641
GU119183	GU119372	GU119326	GU197624
GU119222	GU119287	GU119351	GU197653
GU119211	GU119301	GU119465	GU197623
GU119195	GU119334	GU119478	GU197636
GU119234	GU119299	GU119318	GU197630
GU119189	GU119346	GU119443	GU197640
GU119202	GU119345	GU119338	GU197633
GU119207	GU119379	GU119341	GU197625
GU119252	GU119358	GU119477	GU197635
GU119219	GU119400	GU119489	GU197648
GU119258	GU119337	GU119332	GU197642
GU119200	GU119357	GU119469	GU197652
GU119193	GU119391	GU119408	GU197645
GU119247	GU119370	GU119342	GU197637
GU119231	GU119403	GU119344	GU197647
GU119270	GU119242	GU119481	GU197660
GU119276	GU119415	GU119365	GU197654
GU119268	GU119394	GU119479	GU197657
GU119191	GU119436	GU119374	GU197649
GU119238	GU119236	GU119377	GU197659
GU119282	GU119373	GU119366	GU197661
GU119288	GU119257	GU119398	GU197603

Table S1. *Cont.*

Accession number	Accession number	Accession number	Accession number
GU197639	HM057799	HM129533	HM129566
GU197651	HM105583	HM129572	HM129701
GU197602	HM127314	HM129584	HM129713
GU197638	HM127366	HM129607	HM129567
GU197650	HM127413	HM129601	HM129627
GU197662	HM127423	HM129586	HM129714
GU230462	HM127438	HM129633	HM129807
GU230463	HM127418	HM129686	HM129918
GU230464	HM127444	HM129670	HM129931
GU230461	HM127443	HM129710	HM129943
GU305759	HM127486	HM129634	HM129986
GU305729	HM127422	HM129706	HM151385
GU305793	HM127492	HM129732	HM151383
GU305743	HM127548	HM129757	HM217048
GU305816	HM127546	HM129742	HM217058
GU305837	HM127585	HM129743	HM217055
GU434227	HM127416	HM129751	HM217054
GU437365	HM127437	HM129752	HM217049
GU451376	HM127469	HM129803	HM217065
GU451375	HM127576	HM129753	HM217047
GU451377	HM127600	HM129799	HM217050
GU451432	HM127634	HM129800	HM217059
GU559842	HM127657	HM129811	HM217051
GU810186	HM127772	HM129802	HM217052
GU935362	HM127763	HM129868	HM217069
GU935364	HM127693	HM129902	HM217081
GU935350	HM127660	HM129914	HM224419
GU935366	HM127635	HM129865	HM224444
GU935365	HM127830	HM129939	HM240876
GU935363	HM127671	HM129946	HM240902
GU940676	HM127699	HM129935	HM240879
GU940770	HM127821	HM129960	HM240869
GU940794	HM127784	HM129954	HM240930
GU940818	HM127815	HM129996	HM240887
GU940905	HM127839	HM129797	HM240941
GU940785	HM128955	HM129966	HM240943
GU940825	HM129170	HM129922	HM240872
GU940832	HM127474	HM129829	HM240896
GU941117	HM129215	HM129786	HM240975
GU941146	HM129360	HM130044	HM240981
GU941142	HM129306	HM129940	HM240991
HM057641	HM129435	HM129896	HM240986
HM057711	HM129490	HM129933	HM241013
HM057705	HM129439	HM129964	HM240972
HM057736	HM129565	HM131966	HM240984

Table S1. *Cont.*

Accession number	Accession number	Accession number	Accession number
HM240939	HQ188991	HQ189018	HQ189105
HM240953	HQ188984	HQ189046	HQ189109
HM240968	HQ189005	HQ189079	HQ189117
HM240971	HQ189014	HQ189015	HQ189104
HM241039	HQ189004	HQ189037	HQ189116
HM241030	HQ188996	HQ189078	HQ233045
HM241018	HQ189017	HQ189071	HQ233043
HM241043	HQ189026	HQ189038	HQ233038
HM241035	HQ189016	HQ189045	HQ233039
HM241099	HQ189009	HQ189022	HQ233041
HM346184	HQ188982	HQ189058	HQ233044
HM346183	HQ189031	HQ189029	HQ233042
HM446088	HQ188986	HQ189049	HQ233040
HM573459	HQ189028	HQ189088	HQ241952
HM623781	HQ189030	HQ189099	HQ242023
HM636646	HQ189021	HQ189083	HQ241944
HM636645	HQ188987	HQ189050	HQ242027
HM751855	HQ188994	HQ189057	HQ242036
HM856465	HQ188981	HQ189036	HQ242065
HM856503	HQ189043	HQ189044	HQ242067
HQ008226	HQ188990	HQ189070	HQ242120
HQ008227	HQ189040	HQ189041	HQ242146
HQ008228	HQ189051	HQ189061	HQ242107
HQ157685	HQ189042	HQ189101	HQ242168
HQ157697	HQ189035	HQ189100	HQ242143
HQ157689	HQ188999	HQ189062	HQ242186
HQ157688	HQ189007	HQ189069	HQ242208
HQ157686	HQ188985	HQ189048	HQ242172
HQ157690	HQ188993	HQ189056	HQ242228
HQ157696	HQ189055	HQ189082	HQ242232
HQ157694	HQ189011	HQ189053	HQ242204
HQ157700	HQ189063	HQ189073	HQ242217
HQ157692	HQ189054	HQ189112	HQ242231
HQ157687	HQ189047	HQ189108	HQ242207
HQ157695	HQ189012	HQ189074	HQ242197
HQ157693	HQ188997	HQ189060	HQ242229
HQ157691	HQ189006	HQ189085	HQ242252
HQ157699	HQ189034	HQ189072	HQ242226
HQ166734	HQ189067	HQ189077	HQ242233
HQ166810	HQ189003	HQ189097	HQ242282
HQ189000	HQ189023	HQ189106	HQ242276
HQ189013	HQ189064	HQ189092	HQ242235
HQ189039	HQ189066	HQ189089	HQ242288
HQ188989	HQ189024	HQ189110	HQ242307
HQ189001	HQ189033	HQ189111	HQ242310

Table S1. *Cont.*

Accession number	Accession number	Accession number	Accession number
HQ242326	HQ327233	HQ661209	HQ672079
HQ242271	HQ327212	HQ661201	HQ672091
HQ242290	HQ327267	HQ661206	HQ672138
HQ242323	HQ330627	HQ661254	HQ672151
HQ242272	HQ380799	HQ661274	HQ672104
HQ242320	HQ397170	HQ661291	HQ672145
HQ242338	HQ397201	HQ661192	HQ672148
HQ242335	HQ407328	HQ661203	HQ672140
HQ242303	HQ407323	HQ661359	HQ672160
HQ242347	HQ407326	HQ661253	HQ672175
HQ242325	HQ407322	HQ661262	HQ672193
HQ242308	HQ407325	HQ661318	HQ672207
HQ242333	HQ407324	HQ661325	HQ672224
HQ242328	HQ407327	HQ661357	HQ672227
HQ242441	HQ407329	HQ671750	HQ672232
HQ242453	HQ419032	HQ671783	HQ673362
HQ242446	HQ433564	HQ671775	HQ674060
HQ242410	HQ433561	HQ671804	HQ674063
HQ242477	HQ591517	HQ671797	HQ674488
HQ242490	HQ591529	HQ671796	HQ687069
HQ242469	HQ591511	HQ671766	HQ700837
HQ242455	HQ591512	HQ671827	HQ700832
HQ242488	HQ591524	HQ671801	HQ700834
HQ242456	HQ591515	HQ671856	HQ700836
HQ242450	HQ591509	HQ671802	HQ700838
HQ242474	HQ591516	HQ671836	HQ730085
HQ242449	HQ591513	HQ671854	HQ730899
HQ242447	HQ591522	HQ671878	HQ754549
HQ242468	HQ591528	HQ671874	HQ755632
HQ242467	HQ591526	HQ671871	HQ827853
HQ242478	HQ591520	HQ671901	HQ827865
HQ242480	HQ591521	HQ671917	HQ827877
HQ242473	HQ591510	HQ671909	HQ827849
HQ242491	HQ591533	HQ671925	HQ827852
HQ242471	HQ591523	HQ671928	HQ827845
HQ242476	HQ591518	HQ671950	HQ827861
HQ242472	HQ591514	HQ671973	HQ827846
HQ242484	HQ591519	HQ671989	HQ827864
HQ259629	HQ591530	HQ671997	HQ827855
HQ270352	HQ591527	HQ672020	HQ827851
HQ270311	HQ591531	HQ671936	HQ827854
HQ270357	HQ591532	HQ671975	HQ827850
HQ270415	HQ595191	HQ671983	HQ827857
HQ324869	HQ622720	HQ672065	HQ827873
HQ324870	HQ661169	HQ672056	HQ827858

Table S1. *Cont.*

Accession number	Accession number	Accession number	Accession number
HQ827876	HQ860497	HQ904168	JF703679
HQ827867	HQ860505	HQ904119	JF703683
HQ827856	HQ860512	HQ904142	JF733394
HQ827869	HQ860495	HQ904131	JF733415
HQ827870	HQ860494	HQ904177	JF733399
HQ827880	HQ860510	HQ904175	JF733432
HQ827875	HQ860503	HQ904158	JF747922
HQ827868	HQ860509	HQ904166	JF768745
HQ827878	HQ860507	HQ904169	JF768742
HQ827882	HQ860543	HQ904187	JF768744
HQ827898	HQ860563	HQ904170	JF768743
HQ827901	HQ859461	HQ904178	JF776916
HQ827848	HQ860661	HQ904181	JF776940
HQ827888	HQ860500	HQ904179	JF824768
HQ827881	HQ860496	HQ910311	JF830142
HQ827879	HQ860508	HQ910270	JF830151
HQ827887	HQ904115	HQ912976	JF830199
HQ827894	HQ904141	HQ912984	JF830218
HQ827905	HQ904153	HQ912974	JF830231
HQ827895	HQ904165	HQ912982	JF925013
HQ827860	HQ904127	HQ912979	JF925022
HQ827900	HQ904108	HQ912994	JF966676
HQ827893	HQ904109	HQ912991	JF966677
HQ827899	HQ904123	HQ912990	JF966678
HQ827872	HQ904128	HQ912973	JF966675
HQ827907	HQ904125	HQ912975	JF966674
HQ827847	HQ904140	HQ912989	JN018651
HQ827906	HQ904110	HQ912988	JN018684
HQ827885	HQ904135	HQ912981	JN018665
HQ827859	HQ904152	HQ912983	JN018687
HQ827897	HQ904149	HQ912993	JN018735
HQ827871	HQ904164	HQ912978	JN018715
HQ827904	HQ904126	HQ912995	JN018740
HQ827884	HQ904124	HQ912986	JN018647
HQ827896	HQ904176	HQ912977	JN018650
HQ827903	HQ904138	HQ912985	JN018686
HQ832914	HQ904148	HQ912972	JN018655
HQ832918	HQ904106	HQ912987	JN018664
HQ847580	HQ904162	HQ912980	JN018710
HQ847572	HQ904118	HQ912992	JN018683
HQ847571	HQ904174	JF303683	JN018685
HQ847581	HQ904172	JF417808	JN018713
HQ859462	HQ904132	JF428826	JN018688
HQ859458	HQ904144	JF697566	JN018714
HQ859456	HQ904104	JF703680	JN020217

Table S1. *Cont.*

Accession number	Accession number	Accession number	Accession number
JN032868	JN229902	JN233662	JN547447
JN032869	JN229884	JN257058	JN547441
JN166114	JN229889	JN257070	JN547429
JN166157	JN229948	JN257079	JN547426
JN166126	JN229944	JN257055	JN547453
JN166104	JN229962	JN257067	JN547425
JN166169	JN233000	JN257076	JN547418
JN166181	JN233033	JN257059	JN547433
JN166129	JN233123	JN257071	JN547430
JN166107	JN233163	JN257053	JN547416
JN166150	JN233197	JN257065	JN547445
JN166205	JN233218	JN257060	JN547438
JN166186	JN233234	JN257050	JN547423
JN166146	JN233266	JN257074	JN547428
JN166198	JN233282	JN257072	JN547443
JN166158	JN233276	JN257062	JN547436
JN166127	JN233301	JN257056	JN547419
JN166113	JN233341	JN257052	JN547455
JN166156	JN233288	JN257068	JN547448
JN166163	JN233248	JN257051	JN547431
JN166168	JN233365	JN257057	JN547424
JN166214	JN233262	JN257064	JN547439
JN166180	JN233355	JN257077	JN547451
JN166149	JN233380	JN257063	JN547456
JN166116	JN233347	JN257069	JN547434
JN166152	JN233357	JN257073	JN547437
JN166246	JN233437	JN257054	JN547446
JN166164	JN233349	JN257078	JN547449
JN166142	JN233444	JN257066	JN596604
JN166229	JN233455	JN257048	JN596596
JN166203	JN233415	JN257075	JN596629
JN166237	JN233467	JN257049	JN596598
JN166166	JN233491	JN257061	JN596608
JN166249	JN233376	JN547422	JN596712
JN166257	JN233490	JN547442	JN596634
JN166262	JN233509	JN547454	JN609321
JN166221	JN233575	JN547420	JN655213
JN166220	JN233587	JN547415	JN656759
JN166274	JN233545	JN547432	JN656821
JN166223	JN233640	JN547427	JN656820
JN166231	JN233582	JN547440	JN656774
JN178405	JN233594	JN547435	JN656827
JN178331	JN233664	JN547421	JN656856
JN178673	JN233648	JN547452	JN656803
JN178420	JN233689	JN547417	JN656800

Table S1. *Cont.*

Accession number	Accession number	Accession number	Accession number
JN656816	JN847343	JN874199	JQ237769
JN656873	JN847338	JN874177	JQ237773
JN656822	JN847361	JN874309	JQ237774
JN656854	JN847341	JN874269	JQ237772
JN656866	JN847347	JN874335	JQ237770
JN661705	JN847355	JN874316	JQ259187
JN680690	JN847356	JN874317	JQ326272
JN704797	JN847337	JN874314	JQ323142
JN704799	JN847351	JN874347	L04709
JN704798	JN847354	JN874327	L35331
JN704796	JN847360	JN874325	M63813
JN825307	JN847350	JN874332	U03403
JN825309	JN868821	JN874384	M63814
JN825308	JN868803	JN874364	U03402
JN825341	JN868824	JN874369	U40334
JN825340	JN868846	JN886006	U40338
JN831262	JN868842	JN886018	U40335
JN831261	JN868808	JN886009	U40339
JN831265	JN868853	JN886012	U40331
JN831263	JN868916	JN886013	U40340
JN831264	JN869172	JN886024	U40336
JN847334	JN868978	JN886025	U40337
JN847346	JN869077	JN886011	U40333
JN847359	JN868980	JN886003	U40332
JN847333	JN868986	JN886008	U66194
JN847345	JN869115	JN886015	X52169
JN847358	JN869129	JN886020	X03538
JN847339	JN874178	JN886005	X52171
JN847352	JN874137	JN886007	X70769
JN847336	JN874170	JN886014	X75044
JN847348	JN874130	JN886010	X84811
JN847344	JN874174	JN886019	X84810
JN847340	JN874171	JN886026	X84808
JN847349	JN874162	JN886004	X84809
JN847335	JN874179	JN886016	X99213
JN847357	JN874197	JQ183075	
JN847342	JN874198	JQ183081	
JN847353	JN874208	JQ237768	

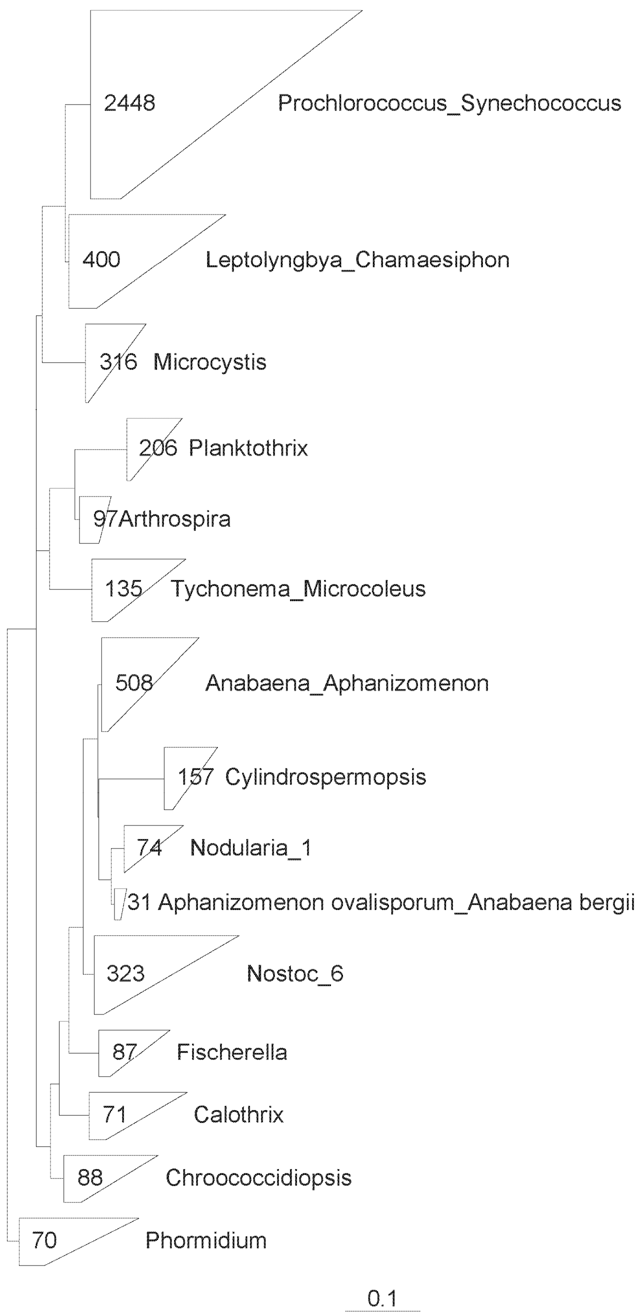

Figure S1. Copy of un-rooted Neighbour Joining tree (Silva release 111) used in this study representing the 15 monophyletic groups chosen for the analysis. Numbers represent the amount of sequences used for each monophyletic clade. The names correspond to the ones from the SILVA database 111 [32].

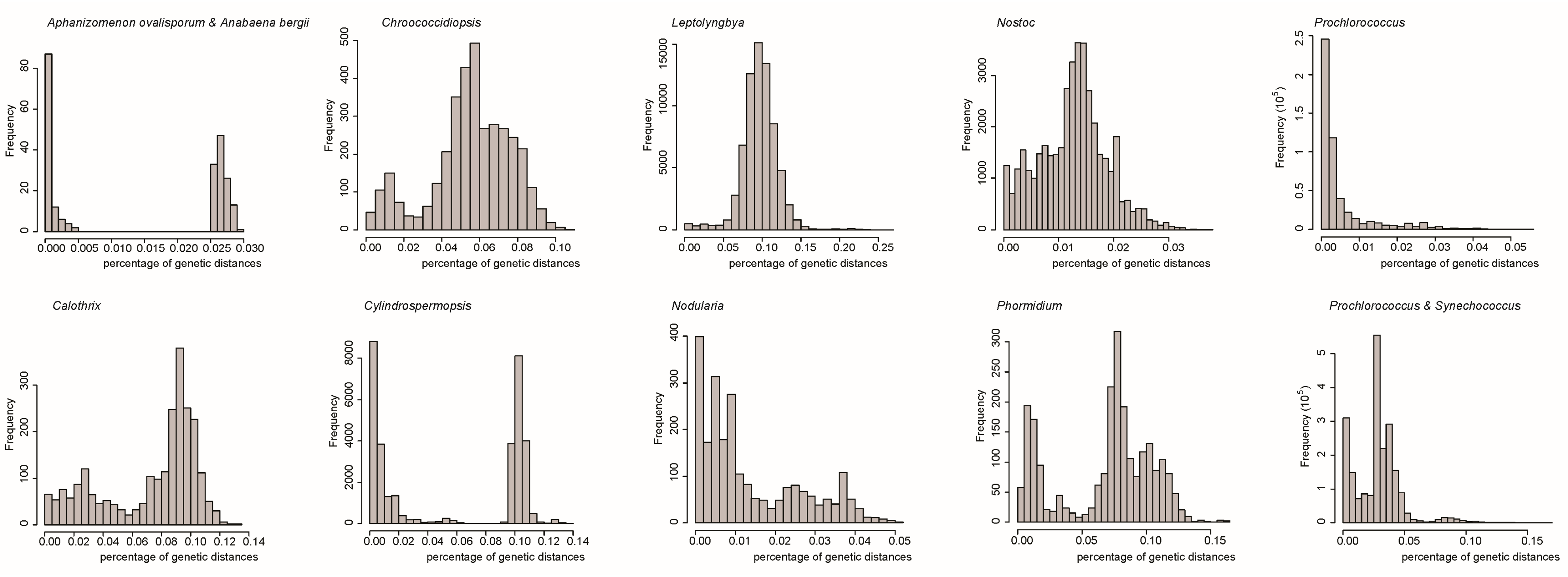

Figure S2. Plot of the distribution of pairwise genetic distances in the ten datasets not depicted in Figure 2. Note the different scale bars of the figures.
